# Effects of a Hydrogel Polymer on the Physiology and Antioxidant Activity of Naturally Colored Cotton Cultivars Under Water Deficit

**DOI:** 10.3390/plants15040667

**Published:** 2026-02-23

**Authors:** Edilene Daniel de Araújo, Lauriane Almeida dos Anjos Soares, Geovani Soares de Lima, Kheila Gomes Nunes, Denis Soares Costa, Allesson Ramos de Souza, Nadiana Praça de Souza, Lucyelly Dâmela Araújo Borborema, Thiago Filipe de Lima Arruda, Francisco de Assis da Silva, André Alisson Rodrigues da Silva, Jailton Garcia Ramos, Viviane Farias Silva, Alberto Soares de Melo, Hans Raj Gheyi, Luciano Marcelo Fallé Saboya

**Affiliations:** 1 Postgraduate Program in Engineering and Management of Natural Resources, Federal University of Campina Grande, Campina Grande 58430-380, Brazil; safirabiologia@gmail.com (E.D.d.A.); jgramos2019@gmail.com (J.G.R.); viviane.farias@professor.ufcg.edu.br (V.F.S.); 2 Academic Unit of Agrarian Sciences, Federal University of Campina Grande, Pombal 58840-000, Brazil; lauriane.soares@professor.ufcg.edu.br; 3 Academic Unit of Agricultural Engineering, Federal University of Campina Grande, Campina Grande 58430-380, Brazil; kheila.gomes@estudante.ufcg.edu.br (K.G.N.); denis.soares@estudante.ufcg.edu.br (D.S.C.); allesson.ramos@estudante.ufcg.edu.br (A.R.d.S.); nadianasouza2018@gmail.com (N.P.d.S.); lucyelly.damela@estudante.ufcg.edu.br (L.D.A.B.); hans.gheyi@ufcg.edu.br (H.R.G.); lsaboya@hotmail.com (L.M.F.S.); 4 Academic Unit of Agronomy, Federal University of Western Pará, Juruti 68170-000, CEP, Brazil; andrealisson_cgpb@hotmail.com; 5 Academic Unit of Agronomy, Federal University of Western Pará, Monte Alegre 68220-000, Brazil; agrofdsilva@gmai.l.com; 6 Department of Biological Sciences, State University of Paraíba, Campina Grande 58429-900, Brazil; alberto.melo@servidor.uepb.edu.br

**Keywords:** *Gossypium hirsutum* L., soil conditioner, abiotic stress, physiological characteristics, agricultural waste

## Abstract

The objective of this study was to evaluate the effects of hydrogel polymer application on the antioxidant activity and physiological performance of colored-fiber cotton cultivars grown under different levels of water restriction. Two experiments were conducted under greenhouse conditions. In the first experiment, the effects of the hydrogel polymer, cultivars, and irrigation replacement levels were evaluated; in the second, the residual effect of the hydrogel polymer applied in the first experiment was assessed using the same cultivars and irrigation depths. Water restriction negatively affected relative water content, gas exchange, chlorophyll *a* fluorescence, and antioxidant activity, and increased electrolyte leakage in cotton cultivars. Water deficit reduced relative water content, gas exchange, chlorophyll *a* fluorescence, and antioxidant activity, while increasing electrolyte leakage in the cultivars. However, hydrogel polymer application up to 6.5 g dm^−3^ of soil and its residual effect in subsequent cycles were beneficial. The polymer increased relative water content and antioxidant activity, in addition to improving gas exchange and chlorophyll fluorescence, suggesting maintenance of plant physiological health. Residual polymer doses also enhanced relative water content, antioxidant activity, gas exchange, and chlorophyll fluorescence in plants during Experiment II.

## 1. Introduction

Herbaceous cotton (*Gossypium hirsutum* L.) is a crop of major global importance, particularly due to its production of natural fiber and vegetable oil, providing livelihoods for approximately 25 million farmers worldwide [1]. However, climate change associated with increasing water scarcity has restricted the growth and productivity of this crop [2], especially in arid and semi-arid regions [3]. The semi-arid region of Northeastern Brazil is characterized by irregular rainfall distribution in both time and space, and when combined with high temperatures, evapotranspiration processes are intensified, exacerbating problems related to water deficit [4,5].

Under water deficit conditions, the reduction in cellular turgor pressure may induce partial stomatal closure [6], decreasing water loss to the atmosphere but limiting CO_2_ diffusion to RuBisCO, thereby reducing the photosynthetic rate [7]. Under these circumstances, increased oxygenation of ribulose−1,5−bisphosphate (RuBP) occurs, favoring the formation of 2-phosphoglycolate and intensifying photorespiration, a process involving the integrated activity of chloroplasts, peroxisomes, and mitochondria, with high energy cost and additional CO_2_ release [8].

In addition, reduced maintenance of plant water status [9] leads to damage to cellular membranes and excessive production of reactive oxygen species (ROS). This process disrupts cellular homeostasis and causes oxidative damage to proteins, DNA, and lipids [10,11]. These species include the superoxide anion (O^2^•^–^), singlet oxygen (^1^O_2_), hydrogen peroxide (H_2_O_2_), and hydroxyl radicals (•OH) [12]. This scenario results in reduced cell expansion [13] and division [14] due to lower water availability within the cellular environment. Overall, water deficit compromises the physiological performance and productivity of agricultural crops [15].

An increase in chlorophyllase enzyme activity may also occur, promoting chlorophyll photooxidation and causing injury to the antenna complex [16]. As a consequence, there is a greater energy demand for electron transport and increased energy dissipation in the form of fluorescence [17], which reduces ATP and NADPH production. Under these conditions, plants activate physiological defense mechanisms, increasing the levels of soluble proteins, protective osmolytes [18], and antioxidant enzymes [19] in order to maintain osmotic and redox homeostasis.

Although it is well established that water plays a vital role in plant development, the underlying response mechanisms have not yet been fully elucidated [20], particularly due to differences among genetic materials. Drought tolerance in agricultural crops is a complex trait resulting from the interaction of multiple agronomic characteristics, each simultaneously influenced by genetic, environmental, and climatic factors [21]. In this context, evaluating the performance of different cotton genotypes under water deficit conditions is of great importance [22].

In addition to the use of different genetic materials, various strategies have been highlighted to mitigate the impacts of water restriction on plants [23], particularly the application of substances with water-retentive properties [24] that assist in maintaining soil moisture. These materials consist of an insoluble cross-linked polymer network that exhibits significant swelling in water, retaining it within its structure in the soil. This allows the soil to maintain an optimal water potential for plant uptake, since moisture is a critical factor influencing crop growth and productivity [25].

In soil, this polymer has the potential to increase water availability, thereby facilitating water and nutrient uptake by plants [26]. When incorporated into the soil, it can absorb and retain water, gradually releasing it to plant roots, as water is attracted into its three-dimensional network through osmotic pressure due to the compatibility between water molecules and polymer chains [27]. Studies indicate that the application of this biopolymer under water deficit conditions may promote plant homeostasis, mainly due to increased water availability. It may also influence soil chemistry and fertility by enhancing the soil’s cation exchange capacity [28]. Additionally, it improves moisture retention, soil fertility, and biological activity, which together contribute to higher plant survival rates [29], even under abiotic stress conditions. When incorporated into the soil, it may further increase nutrient use efficiency by enabling slow and targeted nutrient release to crops [30].

Studies have reported beneficial effects of polymer application in mitigating abiotic stresses. The use of a water-retentive agent at a rate of 36 kg ha^–1^ increased wheat (*Triticum aestivum* L.) yield under water deficit and salinity conditions [31]. Beneficial effects were also observed in Lemon balm (*Melissa officinalis*) [32] and canola (*Brassica napus* L.) [33], where hydrogel polymer application, by improving plant water status, reduced membrane lipid peroxidation and increased the levels of compatible solutes, thereby promoting better osmotic adjustment and improving plant water balance. In a study with okra (*Abelmoschus esculentus* L.), Trigueiro et al. [34] reported that the application of water-retentive polymers promoted positive effects on plant growth, as well as on fresh mass and average fruit diameter, emphasizing that their use may represent an alternative strategy for growers, particularly in semi-arid regions.

However, there are no reports on the application of water-retentive polymers in different cotton cultivars under deficit irrigation in semi-arid conditions. Therefore, this study was based on the hypothesis that soil application of a water-retentive polymer during the first cotton growing cycle, as well as its residual effect in the subsequent cycle, mitigates the deleterious effects of water restriction on the physiology and biochemistry of naturally colored cotton cultivars, improving physiological parameters and enhancing antioxidant system activity. In this context, the objective of this study was to evaluate the effects of hydrogel polymer application on antioxidant activity and physiological performance of different naturally colored cotton cultivars subjected to deficit irrigation over two consecutive growing cycles.

## 2. Results

### 2.1. Experiment I

The multidimensional space of the original variables was summarized into two principal components (PC_1_ and PC_2_) with eigenvalues λ ≥ 1.0. These eigenvalues, together with the percentage of variation explained by each component, accounted for 72.59% of the total variation, with PC_1_ and PC_2_ explaining 52.13% and 20.46% of the variance in Experiment I, respectively. The interaction among irrigation levels, cultivars, and hydroretentive polymer doses (L × C × H) significantly influenced both principal components (Table 1).

**Figure 1:**
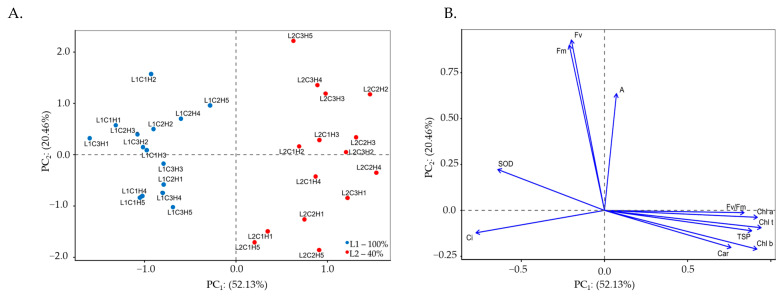
Two-dimensional projection of the treatments (**A**) and the variables analyzed (**B**) in the two components (PC_1_) and (PC_2_). L1—100% of the crop water requirement (control); H2−1.5 g dm^−3^ of soil; H3−3.5 g dm^−3^ of soil; H4−5.0 g dm^−3^ of soil; H5−6.5 g dm^−3^ of soil; PC_1_−Principal Component 1; PC_2_−Principal Component 2; Chl *a*−Chlorophyll *a* (μg mL^−1^); Chl *b*−Chlorophyll *b* (μg mL^−1^); Chl *t*−Total chlorophyll (μg mL^−1^); Car−Carotenoids (μg mL^−1^); *Ci*−Intercellular CO_2_ concentration (μmol CO_2_ m^−3^ s^−1^); *A*−CO_2_ assimilation rate (μmol CO_2_ m^−2^ s^−1^); Fm−Maximum fluorescence; Fv−Variable fluorescence; Fv/Fm−Maximum quantum efficiency of photosystem II (PSII); TSP−Total soluble proteins (mg mL^−1^); SOA−Superoxide dismutase activity (U SOD mg protein^−1^).

Only variables presenting correlation coefficients higher than (|r| > 0.60) were retained in the principal component analysis (PCA). The first component comprised eight variables. Significance (*p* ≤ 0.001) was observed in the first principal component for chlorophyll *a*, chlorophyll *b*, total chlorophyll, carotenoids, internal CO_2_ concentration, quantum efficiency of photosystem II, total soluble proteins, and superoxide dismutase activity. Observe a negative correlation with internal carbon concentration (*Ci*; r = −0.77) and superoxide dismutase activity (SOD; r = −0.64). In addition, PC_1_ exhibited positive correlations with chlorophyll *a* content (Chl *a*; r = 0.92), chlorophyll *b* content (Chl *b*; r = 0.92), total chlorophyll (Chl *t*; r = 0.94), carotenoids (Car; r = 0.76), and the maximum quantum efficiency of photosystem II (Fv/Fm; r = 0.84). The second component was composed of the remaining variables (*p* ≤ 0.001) and showed positive correlations with the CO_2_ assimilation rate (*A*; r = 0.64), maximum fluorescence (Fm; r = 0.90), and variable fluorescence (Fv; r = 0.93). The correlation coefficients among all analyzed variables are presented in Table 1. According to the two-dimensional projection (Figure 1A,B), the variables of naturally colored cotton were separated according to irrigation levels (L1 and L2), with the formation of clusters observed in both components.

In the first component (PC_1_), a potential process characterized by the interaction among irrigation levels (L), cotton cultivars (C), and different concentrations of the hydroretentive polymer (H) was identified. Chlorophyll *a* and carotenoid contents were higher in the cultivar BRS Jade when irrigated with 40% of the crop water requirement and supplied with 5.0 g dm^−3^ of soil (L_2_C_2_H_4_), reaching values of 1999.50 and 398.53 μg mL^−1^, respectively. Compared with plants of the same cultivar under water deficit without hydroretentive polymer application (L_2_C_2_H_1_), increases of 30.16% and 41.41% were observed for Chl *a* and Car, respectively (Table 2).

In cv. BRS Rubi, the highest biopolymer concentration (6.5 g dm^−3^) mitigated the deleterious effects of deficit irrigation (L_2_C_1_H_5_) on Chl *a* content, promoting an increase of 20.56% compared with plants of the same cultivar grown under the same irrigation level without polymer application (L_2_C_1_H_1_). Carotenoid contents in BRS Rubi cotton plants subjected to deficit irrigation increased up to a concentration of 3.5 g dm^−3^, followed by reductions at higher concentrations.

Hydrogel concentrations of 1.5 and 3.5 g dm^−3^ of soil increased chlorophyll *b* and total chlorophyll contents by 31.25% and 29.29%, respectively, in cotton cv. BRS Jade when irrigated with 40% of the crop water requirement (L_2_C_2_H_2_ and L_2_C_2_H_3_), compared with plants of the same cultivar under deficit irrigation without hydrogel application (L_2_C_2_H_1_). However, the highest hydroretentive polymer dose (6.5 g dm^−3^ of soil) reduced chlorophyll *b* and total chlorophyll contents in cv. BRS Verde under deficit irrigation (L_2_C_3_H_5_), with values of 629.18 and 2032.32 μg mL^−1^, resulting in reductions of 31.18% and 26.33%, respectively, compared with plants subjected to the same irrigation level without hydrogel application (L_2_C_3_H_1_).

Intercellular CO_2_ concentration (*Ci*) reached its highest value of 280 μmol CO_2_ m^−2^ s^−1^ under full irrigation in cv. BRS Verde without hydroretentive polymer application (L_1_C_3_H_1_), being 46.34% higher than that observed in plants irrigated with 40% of the crop water requirement without hydrogel application (L_2_C_3_H_1_). Conversely, the hydrogel dose of 6.5 g dm^−3^ resulted in the lowest *Ci* value (159.67 μmol CO_2_ m^−2^ s^−1^) in plants of the same cultivar under deficit irrigation (L_2_C_3_H_5_), corresponding to a reduction of 16.55% compared with the L_2_C_3_H_1_ treatment. Under deficit irrigation (40% of the crop water requirement), the hydroretentive polymer reduced intercellular CO_2_ concentration in cv. BRS Rubi when applied at doses up to 3.5 g dm^−3^ of soil, whereas it increased *Ci* in cv. BRS Jade regardless of the hydroretentive polymer concentration applied.

In the comparison of relative means from Experiment I (Table 2), hydrogel concentrations of 3.5 and 1.5 g dm^−3^ of soil induced higher maximum quantum efficiency of photosystem II and greater total soluble protein content in cotton cv. BRS Verde when irrigated with a deficit irrigation level of 40% (L_2_C_3_H_3_ and L_2_C_3_H_2_), reaching the highest values of Fv/Fm (0.81) and TSP (2.27 mg mL^−1^), respectively. These values corresponded to increases of 0.50% and 36.11% compared with plants grown under the same water conditions without hydrogel application (L_2_C_3_H_1_) for the respective variables. However, under full irrigation, a reduction in the maximum quantum efficiency of photosystem II was observed as a function of hydroretentive polymer application, regardless of the cultivar, with the lowest value (0.778) recorded in cv. BRS Verde under the application of 1.5 g dm^−3^ of soil (L_1_C_3_H_2_).

Superoxide dismutase (SOD) activity increased in cv. BRS Verde under full irrigation without hydrogel application (L_1_C_3_H_1_), reaching 84.41 U SOD mg^−1^ protein, a value 99.28% higher than that observed in plants of the same cultivar grown under deficit irrigation without polymer application (L_2_C_3_H_1_). In contrast, for cultivars BRS Jade and BRS Rubi, SOD activity decreased under cultivation with 100% of the crop water requirement as hydrogel concentrations increased.

The dose of 6.5 g dm^−3^ of soil increased SOD activity in cotton cv. BRS Rubi under deficit irrigation (L_2_C_1_H_5_), reaching 78.15 U SOD mg^−1^ protein, which represents an increase of 179.91% compared with plants grown under the same irrigation condition without hydroretentive polymer application (L_2_C_1_H_1_). However, under deficit irrigation, the lowest SOD activity was recorded in cv. BRS Jade at 6.5 g dm^−3^ of hydroretentive polymer (L_2_C_2_H_5_), with a value of 26.98 U SOD mg^−1^ protein, corresponding to a reduction of 9.64% relative to plants without polymer application under the same water condition (L_2_C_2_H_1_).

Regarding the second component (PC_2_), the CO_2_ assimilation rate of cotton cv. BRS Rubi under full irrigation decreased as a function of increasing hydrogel concentrations. However, application of the highest hydrogel dose increased the net CO_2_ assimilation rate of cotton cv. BRS Verde under deficit irrigation (L_2_C_3_H_5_), reaching 23.26 μmol CO_2_ m^−2^ s^−1^, which represents an increase of 176.90% compared with plants of the same cultivar grown under the same water conditions without hydroretentive polymer application (L_2_C_3_H_1_), which showed the lowest value (8.40 μmol CO_2_ m^−2^ s^−1^). Similar results were observed for cv. BRS Jade irrigated with 40% of the crop water requirement, in which plants treated with 3.5 g dm^−3^ of soil (L_2_C_2_H_3_) exhibited increases of 72.14% relative to the control treatment (L_2_C_2_H_1_).

Deficit irrigation negatively affected maximum and variable fluorescence in the cotton cultivars. However, increasing biopolymer doses promoted increments in these variables in cv. BRS Verde, with Fm and Fv values of 1200 and 946, respectively, when plants were irrigated with 40% of the crop water requirement and treated with 6.5 g dm^−3^ of the biopolymer (L_2_C_3_H_5_). This increase resulted in values 8.70% higher for Fm and 9.11% higher for Fv compared with plants maintained under the same water conditions but without biopolymer application (L_2_C_3_H_1_). Conversely, the highest recorded values of Fm (1228) and Fv (980) were observed in plants of cv. BRS Rubi under full irrigation and treated with 1.5 g dm^−3^ of the biopolymer (L_1_C_1_H_2_).

A significant effect of the interaction among irrigation levels, cultivars, and hydroretentive polymer was observed on relative water content (RWC), catalase (CAT), ascorbate peroxidase (APX), transpiration (*E*), and minimum fluorescence (F_o_) (*p* ≤ 0.01 and 0.05) (Table 3). The EL was influenced by the interaction between irrigation levels and cultivars, as well as between irrigation levels and hydroretentive polymer (*p* ≤ 0.01), respectively. When considered individually, cultivars and irrigation levels significantly affected *gs* (*p* ≤ 0.01) at 75 days after sowing.

**Figure 2:**
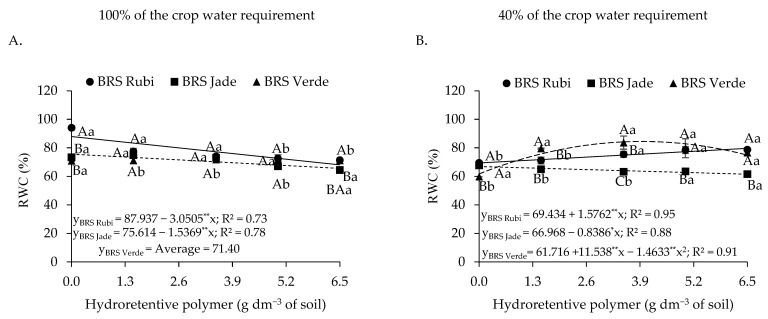
Relative water content—RWC (**A**,**B**) of cotton cultivars under irrigation levels (100 and 40% of the crop water requirement) and hydroretentive polymer application at 75 days after sowing in Experiment I. Identical uppercase letters indicate no significant differences among the different cultivars (Tukey test, *p* ≤ 0.05), whereas identical lowercase letters indicate no significant differences among hydrogel polymer doses (Scott–Knott test, *p* ≤ 0.05). **, and * indicate not significant, significant at *p* ≤ 0.01, and significant at *p* ≤ 0.05, respectively. Vertical bars represent the standard error of the mean (*n* = 3).

Regarding the relative water content of plants under full irrigation (Figure 2A), linear decreases of 3.05% and 1.54% per unit increase in hydroretentive polymer dose were observed for cvs. BRS Rubi and BRS Jade, respectively. For cultivars irrigated with 40% of the crop water requirement (Figure 2B), cv. BRS Rubi showed a linear increase of 1.58% in RWC for each unit increase in hydrogel application. In contrast, cv. BRS Jade exhibited a reduction of 0.84% in RWC per additional unit of hydroretentive polymer. However, an opposite response was observed in cotton cv. BRS Verde, for which a quadratic fit to hydrogel doses indicated an increase of 22.74% at the estimated dose of 3.9 g dm^−3^ of soil compared with the control. Above this dose, a decline in relative water content was observed.

**Figure 3:**
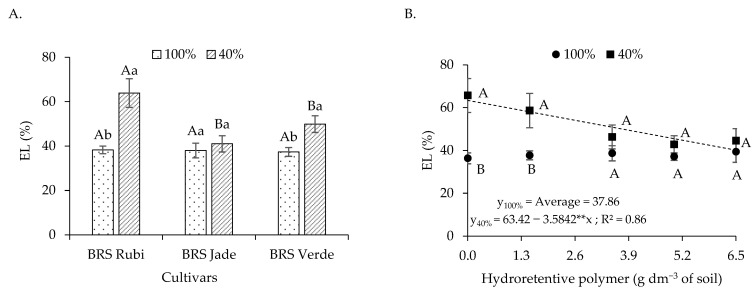
Electrolyte leakage—EL (**A**,**B**) of cotton cultivars under irrigation levels (100 and 40% of the crop water requirement) and hydro-retentive polymer application at 75 days after sowing in Experiment I. Identical uppercase letters indicate no significant differences among the different cultivars (Tukey test, *p* ≤ 0.05), whereas identical lowercase letters indicate no significant differences among hydrogel polymer doses (Scott–Knott test, *p* ≤ 0.05). ** significant at *p* ≤ 0.01. Vertical bars represent the standard error of the mean (*n* = 3).

In plants grown under full irrigation, differences were observed only at doses of 0 and 6.5 g dm^−3^ of soil, in which cv. BRS Rubi increased RWC by 16.30% and 3.79%, respectively, compared with cv. BRS Jade. Under deficit irrigation, cv. BRS Verde showed superiority at doses of 1.5 and 3.5 g dm^−3^, with RWC values of 75.73% and 84.17%, respectively. Cv. BRS Rubi exhibited an increase in RWC as a function of hydrogel doses, whereas cv. BRS Jade showed a reduction (Figure 3B). Overall, when comparing irrigation levels, higher water contents were found under full irrigation. However, application of 5.0 and 6.5 g dm^−3^ in cv. BRS Rubi under water restriction increased RWC by 6.37% and 16.99%, respectively, compared with plants grown under full irrigation subjected to the same treatments.

Regarding electrolyte leakage (Figure 3A), based on the interaction between irrigation levels and naturally colored cotton cultivars, no differences among cultivars were observed under irrigation at 100% of the crop water requirement. When analyzing the effect of irrigation levels within each cultivar, differences were detected only for cvs. BRS Rubi and BRS Verde. The highest electrolyte leakage value (63.88%) was observed in cv. BRS Rubi under deficit irrigation, which was 25.61% higher than that recorded for plants of the same cultivar under full irrigation. Although cv. BRS Verde exhibited lower values than those observed in cv. BRS Rubi under irrigation at 40% of the crop water requirement, electrolyte leakage was still higher (49.90%) compared with the control of the same cultivar (37.37%).

For plants grown under full irrigation and fitted to the regression model (Y_100%_ = 36.748 + 0.3364 ** x) for electrolyte leakage (Figure 3B), no satisfactory adjustment was observed, as the model showed a low coefficient of determination (R^2^ < 0.60). However, under deficit irrigation, a reduction of 5.65% in electrolyte leakage was observed for each unit increase in hydroretentive polymer dose, representing a decrease of 23.29% when comparing plants treated with 0 and 6.5 g dm^−3^ of soil.

**Figure 4:**
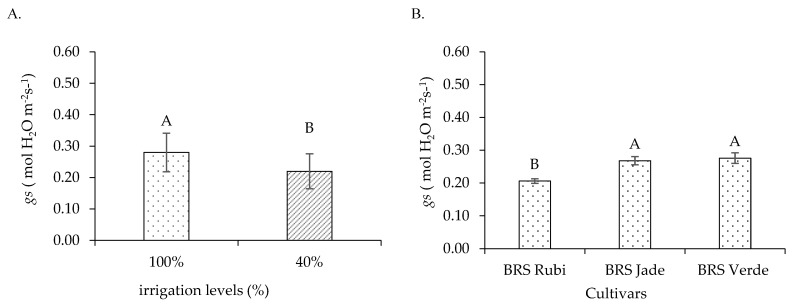
Stomatal conductance—*gs* (**A**,**B**) of naturally colored cotton under irrigation levels (100 and 40% of the crop water requirement) and hydroretentive polymer application at 75 days after sowing in Experiment I. Means followed by the same lowercase and uppercase letters do not differ among cultivars and irrigation levels according to Tukey’s test (*p* ≤ 0.05). V You can replace it with uppercase vertical bars represent the standard error of the mean (*n* = 3).

Stomatal conductance (Figure 4A) was influenced by irrigation levels, with the highest value (0.280 mol H_2_O m^−2^ s^−1^) observed under full irrigation, which was 27.27% higher than that recorded under deficit irrigation. With respect to cultivars (Figure 4B), cvs. BRS Jade and BRS Verde exhibited the highest stomatal conductance values (0.268 and 0.276 mol H_2_O m^−2^ s^−1^, respectively), representing increases of 30.10% and 33.98%, respectively, compared with cv. BRS Rubi, which showed the lowest *gs* value (0.206 mol H_2_O m^−2^ s^−1^).

**Figure 5:**
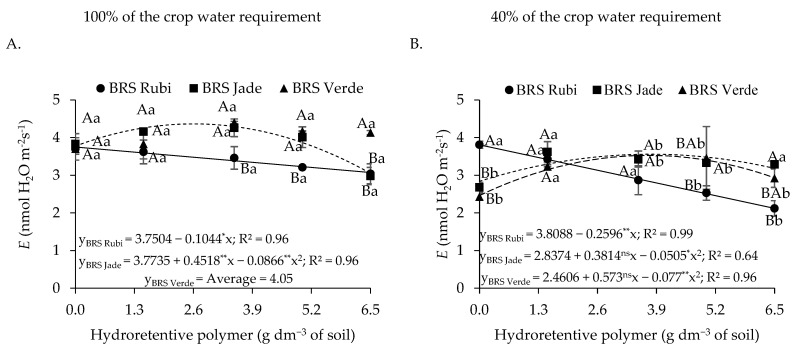
Transpiration—*E* (**A**,**B**) of cotton cultivars under irrigation levels (100 and 40% of the crop water requirement) and hydroretentive polymer application at 75 days after sowing in Experiment I. Identical uppercase letters indicate no significant differences among the different cultivars (Tukey test, *p* ≤ 0.05), whereas identical lowercase letters indicate no significant differences among hydrogel polymer doses (Scott–Knott test, *p* ≤ 0.05). ^ns^, **, and * indicate not significant, significant at *p* ≤ 0.01, and significant at *p* ≤ 0.05, respectively. Vertical bars represent the standard error of the mean (*n* = 3).

For transpiration in plants irrigated with 100% of the crop water requirement (Figure 5A), cv. BRS Rubi showed a linear reduction of 2.78% per unit increase in hydroretentive polymer dose. In contrast, for cv. BRS Jade, the estimated dose of 2.6 g dm^−3^ promoted the highest estimated transpiration (4.36 mmol H_2_O m^−2^ s^−1^), corresponding to an increase of 15.61% compared with the control. Doses above this level reduced transpiration in cv. BRS Jade under full irrigation. Under deficit irrigation (Figure 5B), a reduction of 6.82% per unit increase in hydroretentive polymer dose was observed in the transpiration of cv. BRS Rubi, with a total reduction of 44.30% when comparing the lowest and highest doses. For cvs. BRS Jade and BRS Verde, increases of 25.38% and 43.30% in transpiration, respectively, were observed at the estimated dose of 3.8 g dm^−3^ of soil, compared with plants grown under the same water conditions without hydroretentive polymer application.

When comparing the effects of irrigation levels within each cultivar and hydroretentive polymer dose, plants subjected to irrigation at 100% of the crop water requirement generally showed superior performance (Figure 5A,B). Notably, cv. BRS Jade under full irrigation and a dose of 3.5 g dm^−3^ of soil exhibited an increase of 20.65% compared with plants grown under deficit irrigation at the same polymer concentration. Conversely, cotton cv. BRS Jade irrigated with 40% of the crop water requirement and treated with 6.5 g dm^−3^ of soil showed higher transpiration values (3.18 mmol H_2_O m^−2^ s^−1^) than those observed under optimal water conditions (3.03 mmol H_2_O m^−2^ s^−1^), representing an increase of 4.71%.

**Figure 6:**
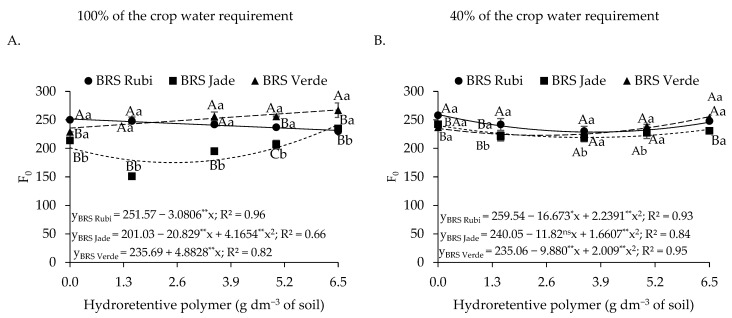
Initial fluorescence—F_0_ (**A**,**B**) of cotton cultivars under irrigation levels (100 and 40% of the crop water requirement) and hydroretentive polymer application at 75 days after sowing in Experiment I. Identical uppercase letters indicate no significant differences among the different cultivars (Tukey test, *p* ≤ 0.05), whereas identical lowercase letters indicate no significant differences among hydrogel polymer doses (Scott–Knott test, *p* ≤ 0.05). ^ns^, **, and * indicate not significant, significant at *p* ≤ 0.01, and significant at *p* ≤ 0.05, respectively. Vertical bars represent the standard error of the mean (*n* = 3).

With respect to initial fluorescence in plants under full irrigation (Figure 6A), cv. BRS Rubi exhibited a linear decrease of 1.22% per unit increase in hydroretentive polymer dose, reaching a reduction of 7.96% at 6.5 g dm^−3^ of soil compared with the control (0 g dm^−3^). An opposite response was observed in cv. BRS Verde, in which an increase of 2.07% per unit of hydrogel was recorded, resulting in an increase of 13.47% at the dose of 6.5 g dm^−3^ of soil compared with plants not treated with the hydroretentive polymer. Cv. BRS Jade showed a quadratic response to hydrogel doses, with a reduction of 12.95% (26.04) at the estimated dose of 2.5 g dm^−3^ of soil relative to the control. Under deficit irrigation (Figure 6B), a similar response was observed for cvs. BRS Rubi and BRS Jade, in which hydroretentive polymer application up to 3.7 g dm^−3^ of soil reduced F_o_, with values of 228.5 and 219.05, corresponding to reductions of 11.95% and 8.74% relative to the control treatment of each cultivar, respectively. In cv. BRS Verde, the dose of 2.5 g dm^−3^ reduced initial fluorescence by 5.16%; however, at concentrations above this level, an increase in F_o_ was observed.

In the interaction among irrigation levels, cultivars, and hydroretentive polymer doses (Figure 6A,B), lower initial fluorescence was observed in plants grown under full irrigation conditions, where a dose of 6.5 g dm^−3^ of soil provided a 5.79% reduction in the cv. BRS Rubi compared to those grown under deficit irrigation. Conversely, when cvs. BRS Jade and BRS Verde were irrigated with 40% of the crop water requirement, a beneficial effect of applying 6.5 g dm^−3^ of the hydroretentive polymer was observed, resulting in reductions of 3.38% and 4.38%, respectively, compared with plants grown under the same conditions but under full irrigation.

**Figure 7:**
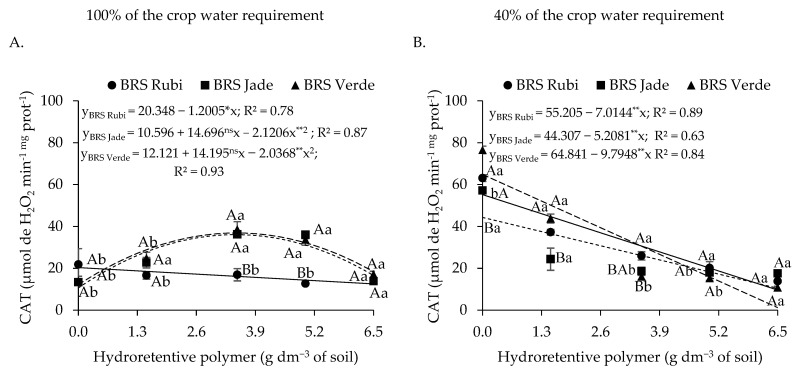
Catalase activity—CAT (**A**,**B**) of cotton cultivars under irrigation levels (100 and 40% of the crop water requirement) and hydroretentive polymer application at 75 days after sowing in Experiment I. Identical uppercase letters indicate no significant differences among the different cultivars (Tukey test, *p* ≤ 0.05), whereas identical lowercase letters indicate no significant differences among hydrogel polymer doses (Scott–Knott test, *p* ≤ 0.05). ^ns^, **, and * indicate not significant, significant at *p* ≤ 0.01, and significant at *p* ≤ 0.05, respectively. Vertical bars represent the standard error of the mean (*n* = 3).

Regarding catalase activity in plants under full irrigation (Figure 7A), cv. BRS Rubi exhibited a reduction of 5.90% per unit increase in hydroretentive polymer dose, with a total reduction of 38.35% (7.80 μmol H_2_O_2_ min^−1^ mg protein^−1^) at the dose of 6.5 g dm^−3^ of soil compared with the control. For cvs. BRS Jade and BRS Verde, CAT activity increased by 240.27% and 204.04%, respectively, at the dose of 3.5 g dm^−3^ compared with 0 g dm^−3^. Doses above this estimated level resulted in a reduction in CAT activity, with decreases of 54.12% and 50.22%, respectively, when compared with the highest evaluated dose. In Figure 7B, plants subjected to deficit irrigation exhibited linear reductions in CAT activity. These reductions corresponded to decreases of 12.71%, 11.75%, and 15.11% per unit increase in hydroretentive polymer dose for cotton cvs. BRS Rubi, BRS Jade, and BRS Verde, respectively, resulting in reductions of 82.59%, 76.40%, and 98.19% at the dose of 6.5 g dm^−3^ compared with the control treatment.

In plants grown under 100% of the crop water requirement, differences among cultivars were observed only at doses of 3.5 and 5.0 g dm^−3^ of soil, in which cvs. BRS Jade and BRS Verde exhibited CAT activity values of 36.06 and 31.06, and 38.85 and 32.17 μmol H_2_O_2_ min^−1^ mg protein^−1^, respectively. These values were 123.30% and 116.52%, and 128.24% and 124.29% higher at the respective doses compared with cv. BRS Rubi. For plants under deficit irrigation, differences were observed only at doses of 0.0, 1.5, and 3.5 g dm^−3^, with the highest CAT activity recorded in cv. BRS Rubi. Overall, higher catalase activity was observed in plants irrigated with 40% of the crop water requirement (Figure 7A,B). However, this response was attenuated by hydroretentive polymer application up to a concentration of 6.5 g dm^−3^ in cvs. BRS Rubi, BRS Jade, and BRS Verde, resulting in reductions of 23.38%, 36.73%, and 93.42%, respectively, compared with plants grown under the same conditions but subjected to full irrigation.

**Figure 8:**
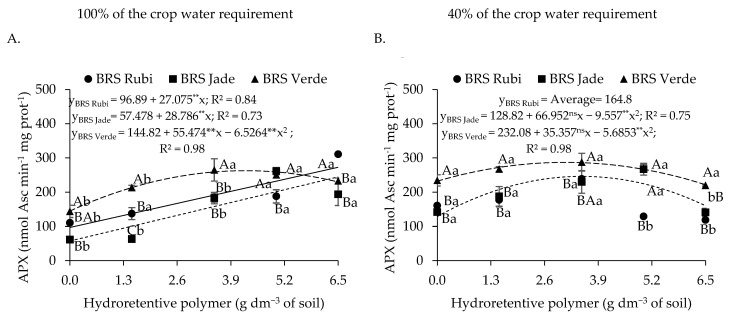
Ascorbate peroxidase activity—APX (**A**,**B**) of cotton cultivars under irrigation levels (100 and 40% of the crop water requirement) and hydroretentive polymer application at 75 days after sowing in Experiment I. Identical uppercase letters indicate no significant differences among the different cultivars (Tukey test, *p* ≤ 0.05), whereas identical lowercase letters indicate no significant differences among hydrogel polymer doses (Scott–Knott test, *p* ≤ 0.05). ^ns^ and ** indicate not significant and significant at *p* ≤ 0.01, respectively. Vertical bars represent the standard error of the mean (*n* = 3).

For ascorbate peroxidase activity in plants irrigated with 100% of the crop water requirement (Figure 8A), cv. BRS Rubi exhibited an increase of 181.64% (175.99 nmol ASC min^−1^ mg protein^−1^) at the dose of 6.5 g dm^−3^ of soil, with a linear increment of 27.94% per unit increase in hydroretentive polymer dose compared with the control. In cvs. BRS Jade and BRS Verde, APX activity increased by 443.82% and 102.64%, corresponding to 179.92 and 117.85 nmol ASC min^−1^ mg protein^−1^ at the estimated doses of 6.3 and 4.2 g dm^−3^ of soil, respectively, compared with the control. Similarly, under deficit irrigation (Figure 8B), increases of 91.03% and 23.69% in APX activity were observed in cvs. BRS Jade and BRS Verde, respectively, at hydrogel doses of 3.5 and 3.1 g dm^−3^ of soil relative to the control. Beyond these doses, reductions in APX activity were observed in the cultivars.

Under both full irrigation (Figure 8A) and deficit irrigation (Figure 8B), cv. BRS Verde exhibited superior APX activity at all hydroretentive polymer doses, except at the dose of 5.0 g dm^−3^ of soil under deficit irrigation (Figure 8B). However, the highest antioxidant activity was observed in cotton plants irrigated with 40% of the crop water requirement. Cv. BRS Verde under deficit irrigation showed APX values of 232.08, 272.32, 286.18, and 266.72 nmol ASC min^−1^ mg protein^−1^, representing increases of 60.25%, 27.64%, 10.48%, and 2.97% at doses of 0, 1.5, 3.5, and 5.0 g dm^−3^, respectively, compared with plants under the same treatments but irrigated at 100% of the crop water requirement. A similar effect was observed in cv. BRS Jade, in which deficit irrigation resulted in increases of 124.12%, 106.39%, 55.52%, and 11.54% at doses of 0, 1.5, 3.5, and 5.0 g dm^−3^, respectively, compared with cotton plants grown under the same treatments but under full irrigation (Figure 8A,B).

### 2.2. Experiment II

With regard to Experiment II, similarly to Experiment I, the original variables were summarized into two principal components with eigenvalues (λ ≥ 1.0). These eigenvalues, together with the percentage of variance explained by each component, accounted for 70.24% of the total variation. Individually, PC_1_ explained 48.10% of the variance, whereas PC_2_ accounted for 22.14%. The interaction among irrigation levels, cultivars, and hydroretentive polymer doses (L × C × H) significantly influenced both principal components (Table 4).

**Figure 9:**
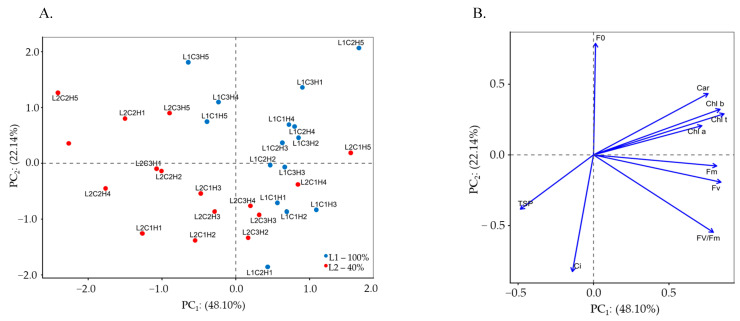
Two-dimensional projection of treatments (**A**) and analyzed variables (**B**) on the two principal components (PC_1_ and PC_2_). L1—100% of the crop water requirement (control); H2−1.5 g dm^−3^ of soil; H3−3.5 g dm^−3^ of soil; H4−5.0 g dm^−3^ of soil; H5−6.5 g dm^−3^ of soil; PC_1_−Principal Component 1; PC_2_−Principal Component 2; Chl *a*—chlorophyll *a* (µg mL^−1^); Chl *b*—chlorophyll *b* (µg mL^−1^); Chl total—total chlorophyll (µg mL^−1^); Car—carotenoids (µg mL^−1^); *Ci*—intercellular CO_2_ concentration (µmol CO_2_ m^−2^ s^−1^); F_o_—initial fluorescence; Fm—maximum fluorescence; Fv—variable fluorescence; Fv/Fm—maximum quantum efficiency of photosystem II; TSP—total soluble proteins (mg mL^−1^).

Variables presenting correlation coefficients higher than 0.60 (|r| > 0.60) were retained in the principal component analysis (PCA), as shown in Table 4. The first component comprised eight variables (*p* ≤ 0.001), showing a positive correlation only for total soluble proteins (TSP; r = 0.63), for chlorophyll *a* (Chl *a*; r = 0.72), chlorophyll *b* (Chl *b*; r = 0.84), total chlorophyll (Chl t; r = 0.87), carotenoids (Car; r = 0.76), maximum fluorescence (Fm; r = 0.87), variable fluorescence (Fv; r = 0.85), and the maximum quantum efficiency of photosystem II (Fv/Fm; r = 0.85) (Figure 9).

**Figure 10:**
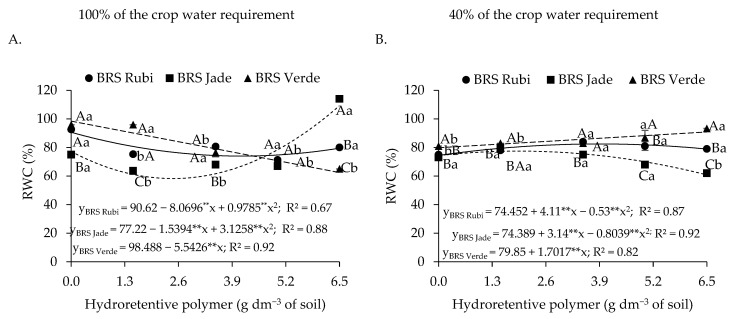
Relative water content—RWC (**A**,**B**) of cotton cultivars under irrigation levels (100 and 40% of the crop water requirement) and hydroretentive polymer application at 77 days after sowing in Experiment II. Identical uppercase letters indicate no significant differences among the different cultivars (Tukey test, *p* ≤ 0.05), whereas identical lowercase letters indicate no significant differences among hydrogel polymer doses (Scott–Knott test, *p* ≤ 0.05). ** indicate significant at *p* ≤ 0.01. Vertical bars represent the standard error of the mean (*n* = 3).

The second component was represented by the remaining variables, showing a negative correlation (*p* ≤ 0.001) with initial fluorescence (F_o_; r = 0.78) and a negative correlation with intercellular CO_2_ concentration (*Ci*; r = −0.83). According to the two-dimensional projection (Figure 10A,B), the cotton variables were separated according to irrigation levels (L1 = 100% of the crop water requirement and L2 = 40% of the crop water requirement).

Overall, higher values of photosynthetic pigment contents were observed in cotton plants irrigated with 100% of the crop water requirement (Table 5). Under full irrigation, cv. BRS Jade grown under the residual effect of the 6.5 g dm^−3^ soil dose (L_1_C_2_H_5_) exhibited the highest values of 1781.32, 1366.28, 3147.60, and 373.05 μg mL^−1^, corresponding to increases of 28.02%, 24.26%, 26.50%, and 26.82% for chlorophyll *a*, chlorophyll *b*, total chlorophyll and carotenoids, respectively, compared with plants of the same cultivar grown under the same irrigation conditions but without residual hydroretentive polymer application (L_1_C_2_H_1_).

However, despite lower values compared with those observed under full irrigation, the residual effect of the hydroretentive polymer promoted increases in pigment contents across different cotton cultivars under deficit irrigation. When cv. BRS Rubi was irrigated with 40% of the crop water requirement under the residual dose of 6.5 g dm^−3^ (L_2_C_1_H_5_), increases of 42.71%, 20.60%, 32.74%, and 45.58% were observed for chlorophyll *a*, chlorophyll *b*, total chlorophyll and carotenoids, respectively, compared with plants grown under the same water conditions but without residual hydrogel application (L_2_C_1_H_1_). Similar results were observed for cv. BRS Jade under the residual dose of 3.5 g dm^−3^ of soil (L_2_C_2_H_3_), which exhibited values of 1655.20, 1043.41, 2689.61 and 283.93 μg mL^−1^, corresponding to increases of 44.32%, 2.86%, 24.86% and 24.76% for chlorophyll *a*, chlorophyll *b*, total chlorophyll and carotenoids, respectively, compared with plants under the same water status but without polymer residue (L_2_C_2_H_1_).

Although higher photosynthetic pigment contents were observed in plants under full irrigation, cotton cv. BRS Rubi grown under deficit irrigation with the residual dose of 6.5 g dm^−3^ (L_2_C_1_H_5_) exhibited the highest values of maximum fluorescence (Fm), variable fluorescence (Fv), and maximum quantum efficiency of photosystem II (Fv/Fm), reaching 1284, 1046 and 0.827, respectively. These values represent increases of 17.91%, 21.63% and 4.87%, respectively, compared with plants grown under the same water conditions without residual hydroretentive polymer application (L_2_C_1_H_1_), indicating that despite the reduction in pigment contents, the photosynthetic apparatus remained intact. A similar effect was observed for cv. BRS Verde, in which the residual dose of 3.5 g dm^−3^ (L_2_C_3_H_3_) increased Fm by 11.42% and 15.18% for Fm and Fv, respectively, in comparison with plants under the same water conditions but under 0 g dm^−3^ of residue (L_2_C_3_H_1_). Regarding the quantum efficiency of photosystem II, the L_2_C_3_H_2_ treatment increased the Fm/Fv ratio by 3.79% compared to the L_2_C_3_H_1_ treatment.

Conversely, in the comparison of relative means from Experiment II (Table 5), cv. BRS Jade irrigated with 40% of the crop water requirement under the residual effect of the highest biopolymer dose (6.5 g dm^−3^ of soil) (L_2_C_2_H_5_) exhibited the lowest values of maximum fluorescence (Fm), variable fluorescence (Fv), and maximum quantum efficiency of photosystem II (Fv/Fm), with values of 961, 728.33, and 0.758, respectively.

Still within the first component, total soluble proteins (TSP) were influenced by the interaction among the analyzed factors (L × C × H). Under full irrigation, reductions in TSP were observed in all cultivars, regardless of the residual dose of the hydroretentive polymer. However, the highest mean values were recorded in plants grown under deficit irrigation. When irrigation was applied at 40% of the crop water requirement in cv. BRS Jade under the residual dose of 1.5 g dm^−3^ of soil of the hydroretentive polymer (L_2_C_2_H_2_), the highest TSP value was observed (2.231 mg mL^−1^), representing an increase of 37.80% compared with plants grown under the same conditions but without polymer residue (L_2_C_2_H_1_). Conversely, the lowest mean TSP value was recorded under full irrigation in cv. BRS Verde at the dose of 5.0 g dm^−3^ of soil (L_1_C_3_H_4_), showing a reduction of 53.31% compared with plants subjected to deficit irrigation under the same residual polymer concentration (L_2_C_3_H_4_).

With respect to the second component, the highest intercellular CO_2_ concentration (*Ci*; 282 μmol CO_2_ m^−2^ s^−1^) was recorded in cv. BRS Rubi subjected to deficit irrigation without polymer application (L_2_C_1_H_1_), representing an increase of 12.25% compared with plants grown under full irrigation at the same polymer dose (L_1_C_1_H_1_). Cotton cultivars grown under deficit irrigation without polymer application showed reductions in *Ci* of 22.06% in cv. BRS Jade (L_2_C_2_H_1_) and 12.63% in cv. BRS Verde (L_2_C_3_H_1_) relative to cv. BRS Rubi (L_2_C_1_H_1_). However, the residual dose of 6.5 g dm^−3^ of soil reduced *Ci* by 28.33% in cotton cv. BRS Rubi under deficit irrigation (L_2_C_1_H_5_) compared with treatment L_2_C_1_H_1_. The lowest mean *Ci* value (187.67 μmol CO_2_ m^−2^ s^−1^) was recorded in cv. BRS Verde grown under full irrigation with the residual dose of 6.5 g dm^−3^ of soil (L_1_C_3_H_5_).

Regarding initial fluorescence (F_o_), cv. BRS Jade grown under deficit irrigation without polymer residue (L_2_C_2_H_1_) exhibited the highest mean value (242), representing an increase of 23.47% compared with treatment L_1_C_2_H_1_, which corresponded to full irrigation without hydrogel application and showed the lowest value (196). However, the residual dose of 3.5 g dm^−3^ of soil reduced F_o_ by 17.91% in cv. BRS Jade under water restriction (L_2_C_2_H_3_) relative to treatment L_2_C_2_H_1_. With respect to cultivars, reductions of 10.47% and 6.61% in F_o_ were recorded for cotton cvs. BRS Rubi (L_2_C_1_H_1_) and BRS Verde (L_2_C_3_H_1_), respectively, under deficit irrigation without polymer residue when compared with cv. BRS Jade grown under the same water conditions and residual dose (L_2_C_2_H_1_).

A significant effect (*p* ≤ 0.01) of the interaction L × C × H was observed on relative water content (RWC), electrolyte leakage (EL), superoxide dismutase (SOD), catalase (CAT), ascorbate peroxidase (APX), and stomatal conductance (*gs*) (Table 6). A significant effect (*p* ≤ 0.05) was also detected for the interaction between irrigation levels and hydroretentive polymer doses (L × H) on the CO_2_ assimilation rate (*A*). When analyzed individually, hydroretentive polymer doses had a significant effect (*p* ≤ 0.01) on cotton transpiration (*E*) at 77 days after sowing.

**Figure 11:**
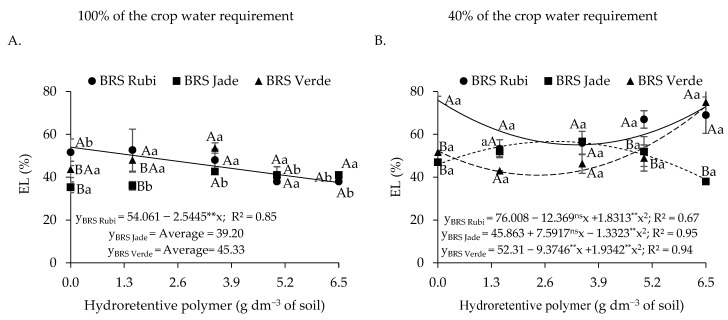
Electrolyte leakage—EL (**A**,**B**) of cotton cultivars under irrigation levels (100 and 40% of the crop water requirement) and hydroretentive polymer application 77 days after sowing in Experiment II. Identical uppercase letters indicate no significant differences among the different cultivars (Tukey test, *p* ≤ 0.05), whereas identical lowercase letters indicate no significant differences among hydrogel polymer doses (Scott–Knott test, *p* ≤ 0.05). ^ns^ and ** indicate not significant and significant at *p* ≤ 0.01, respectively. Vertical bars represent the standard error of the mean (*n* = 3).

For relative water content in plants under full irrigation (Figure 11A), cvs. BRS Rubi and BRS Jade showed reductions of 16.63% and 18.94% at the estimated doses of 4.1 and 2.5 g dm^−3^ of soil, respectively, compared with the control. Above these doses, increases of 5.52% and 50.89% were observed when compared with plants under the residual dose of 6.5 g dm^−3^ of soil. In contrast, cv. BRS Verde exhibited a linear decrease, with a reduction of 5.54% per unit increase in hydroretentive polymer dose. Under deficit irrigation (Figure 11B), residual doses promoted increases in relative water content in all cultivars. However, cvs. BRS Rubi and BRS Jade at the estimated residual doses of 3.9 and 2.0 g dm^−3^ of soil increased RWC by 8.02% and 3.05%, respectively, compared with plants not treated with hydrogel. Cv. BRS Verde showed an increase of 11.06% in RWC at the dose of 6.5 g dm^−3^ of soil compared with plants without polymer residue.

In plants irrigated with 100% of the crop water requirement (Figure 10A), differences among cultivars were observed at all doses evaluated, except for the dose of 5.0 g dm^−3^ of soil. The cv. BRS Verde showed the highest RWC at the dose of 1.5 g dm^−3^. The highest estimated value (109.23%) was recorded in cotton cv. BRS Jade under the residual dose of 6.5 g dm^−3^. In plants irrigated with 40% of the crop water requirement (Figure 10B), differences among cultivars were observed at all hydroretentive polymer doses, with cv. BRS Verde outperforming the others at all doses except 3.5 g dm^−3^ of soil. At doses of 5.0 and 6.5 g dm^−3^, increases of 19.00% and 31.33%, respectively, were observed compared with cv. BRS Jade. Regarding irrigation levels (Figure 10A,B), plants under full irrigation generally exhibited higher relative water content.

However, hydroretentive polymer application showed beneficial effects for cvs. BRS Rubi and BRS Verde only under deficit irrigation. At dose 5.0 g dm^−3^, cv. BRS Rubi exhibited higher RWC values than plants of the same cultivar grown under the same conditions but with full irrigation. A similar effect was observed for cv. BRS Verde, in which residual doses of 3.5, 5.0, and 6.5 g dm^−3^ of soil increased relative water content by 6.69%, 17.56% and 28.42%, respectively, compared with plants grown under the same polymer concentrations but irrigated with 100% of the crop water requirement (Figure 10A,B).

**Figure 12:**
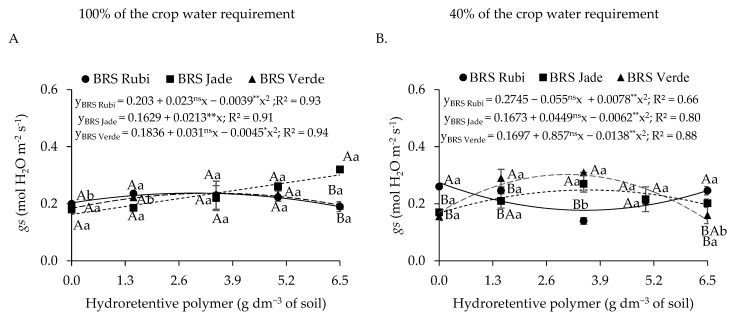
Stomatal conductance—gs (**A**,**B**) of cotton cultivars under irrigation levels (100 and 40% of the crop water requirement) and hydroretentive polymer application at 77 days after sowing in Experiment II. Identical uppercase letters indicate no significant differences among the different cultivars (Tukey test, *p* ≤ 0.05), whereas identical lowercase letters indicate no significant differences among hydrogel polymer doses (Scott–Knott test, *p* ≤ 0.05). ^ns^, **, and * indicate not significant, significant at *p* ≤ 0.01, and significant at *p* ≤ 0.05, respectively. Vertical bars represent the standard error of the mean (*n* = 3).

For electrolyte leakage in cotton cultivars under full irrigation (Figure 11A), the residual dose of 6.5 g dm^−3^ of soil reduced EL by 16.53% in cv. BRS Rubi, corresponding to a reduction of 2.74% per unit increase in hydroretentive polymer dose. Under deficit irrigation (Figure 12B), the estimated residual doses of 3.4 and 2.4 g dm^−3^ of soil reduced EL in cvs. BRS Rubi and BRS Verde by 20.88% and 11.36%, respectively, compared with the control. Conversely, in cv. BRS Jade, an increase of 10.31% electrolyte leakage was observed at the estimated residual dose of 2.8 g dm^−3^ of soil, followed by a reduction of 17.75% beyond this dose relative to the control.

It should be noted that no significant differences among cultivars were observed at the doses of 1.5 and 3.5 g dm^−3^ of soil in plants grown under 40% of the crop water requirement (Figure 11B). However, cotton cv. BRS Rubi recorded the highest electrolyte leakage values at doses of 0 and 5.0 g dm^−3^ of soil, which were statistically higher than those observed in cv. BRS Jade. When examining the interaction among irrigation levels, cultivars, and different residual doses of the hydroretentive polymer (Figure 11A,B), the highest percentage of electrolyte leakage was observed in cv. BRS Rubi under deficit irrigation, with increases of 21.94%, 11.32%, 10.00%, 18.60%, and 35.46% at doses of 0, 1.5, 3.5, 5.0, and 6.5 g dm^−3^ of soil, respectively, compared with plants grown under full irrigation conditions.

Residual doses of the hydroretentive polymer promoted increases in stomatal conductance in plants grown under full irrigation conditions (Figure 12A). In cv. BRS Jade, stomatal conductance increased by 84.99% at the dose of 6.5 g dm^−3^ of soil, corresponding to a unit increase of 13.08% per additional unit of polymer compared with the control. Cvs. BRS Rubi and BRS Verde exhibited the highest *gs* values (0.236 and 0.237 mol H_2_O m^−2^ s^−1^, respectively) when grown under an estimated residual dose of 3.4 g dm^−3^ of soil of the hydroretentive polymer, representing increases of 16.31% and 29.07%, respectively, compared with the control of each cultivar. Regarding plants grown under deficit irrigation (Figure 12B), cv. BRS Rubi showed a reduction of 35.45% in stomatal conductance at the estimated dose of 3.5 g dm^−3^ of soil relative to the control, followed by an increase of 38.75% at doses above this level. In addition, cvs. BRS Jade and BRS Verde exhibited increases of 48.59% and 83.99% in stomatal conductance at the estimated doses of 3.6 and 3.2 g dm^−3^ of soil, respectively, compared with the dose of 0 g dm^−3^ of soil.

Differences among cultivars under full irrigation (Figure 12A) were observed only at the residual dose of 6.5 g dm^−3^ of soil, at which cv. BRS Jade stood out with the highest stomatal conductance value (0.30 mol H_2_O m^−2^ s^−1^), being 60.53% and 54.56% higher than those of cvs. BRS Rubi and BRS Verde, respectively, under the same conditions. Under deficit irrigation, the highest stomatal conductance (*gs*) values were recorded in the BRS Rubi cultivar, with significant differences observed at the doses of 0 and 6.5 g^−3^ dm of soil. Under these conditions, *gs* increased by 64.07% and 24.69% compared with the BRS Jade cultivar and by 61.75% and 71.12% compared with the BRS Verde cultivar at the respective doses. At the dose of 1.5 g dm^−3^ of soil, cv. BRS Verde exhibited superiority, with an increase of 38.10% in stomatal conductance relative to cv. BRS Jade. When irrigation levels were compared (Figure 12A,B), stomatal conductance was higher under full irrigation at the dose of 3.5 g dm^−3^ for cv. BRS Rubi and at the dose of 6.5 g dm^−3^ for cv. BRS Jade, whereas at the dose of 1.5 g dm^−3^ of soil, *gs* was lower for cv. BRS Verde.

**Figure 13:**
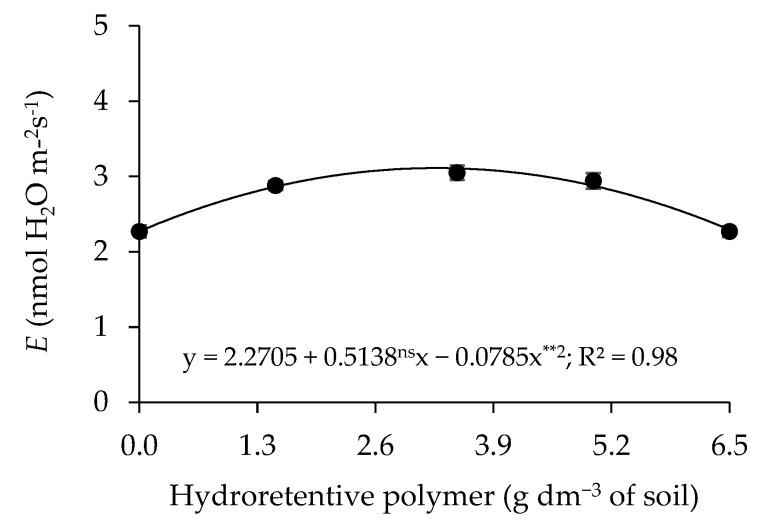
Transpiration—*E* of cotton cultivars under hydroretentive polymer application at 77 days after sowing in Experiment II. ^ns^ and ** indicate not significant and significant at *p* ≤ 0.01, respectively. Vertical bars represent the standard error of the mean (*n* = 3).

Transpiration (Figure 13) was affected quadratically as a function of residual doses of the hydroretentive polymer, with the highest value of 3.11 mmol of H_2_O m^−2^ s^−1^ recorded in plants grown at the estimated residual dose of 3.3 g dm^−3^ of soil, corresponding to increases of 37.03% (0.84 mmol of H_2_O m^−2^ s^−1^) compared to those under a concentration of 0 g dm^−3^ of soil.

**Figure 14:**
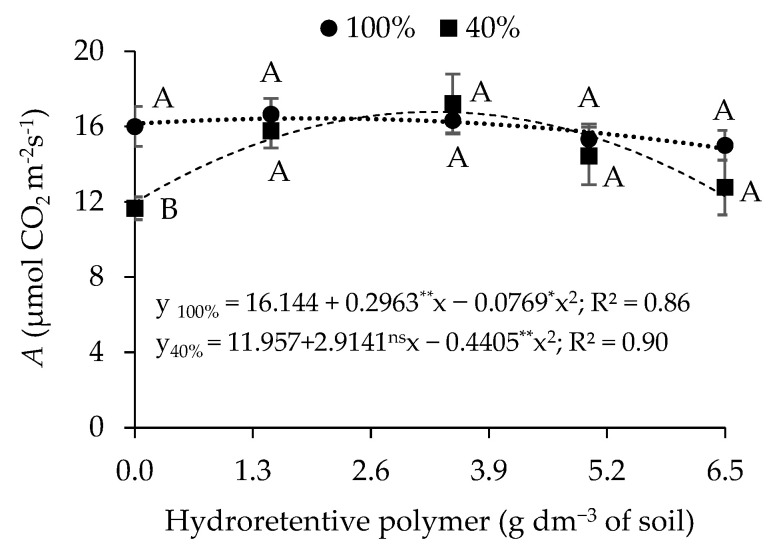
CO_2_ assimilation rate—*A* of cotton cultivars under irrigation levels (100 and 40% of the crop water requirement) and hydroretentive polymer application at 77 days after sowing in Experiment II. Means followed by the same lowercase and uppercase letters do not differ among cultivars and irrigation levels according to Tukey’s test (*p* ≤ 0.05). ^ns^, **, and * indicate not significant, significant at *p* ≤ 0.01, and significant at *p* ≤ 0.05, respectively. Vertical bars represent the standard error of the mean (*n* = 3).

In plants irrigated with 100% of the crop water requirement, a 1.77% increase in CO_2_ assimilation rate was observed (Figure 14) up to the residual dose of 1.9 g dm^−3^ of soil. However, above this dose, a reduction in transpiration (*E*) was observed. In addition, a higher CO_2_ assimilation rate was found in plants irrigated with 40% of the crop water requirement at the estimated residual dose of 3.3 g dm^−3^ of soil, resulting in an increase of 40.31% compared with plants grown under the same water condition but without hydrogel application. However, differences between irrigation levels were observed only in plants grown without hydroretentive polymer application (0 g dm^−3^ of soil), in which the lowest CO_2_ assimilation rate (11.95 μmol CO_2_ m^−2^ s^−1^) was recorded under deficit irrigation, representing a reduction of 25.93% compared with the control treatment.

**Figure 15:**
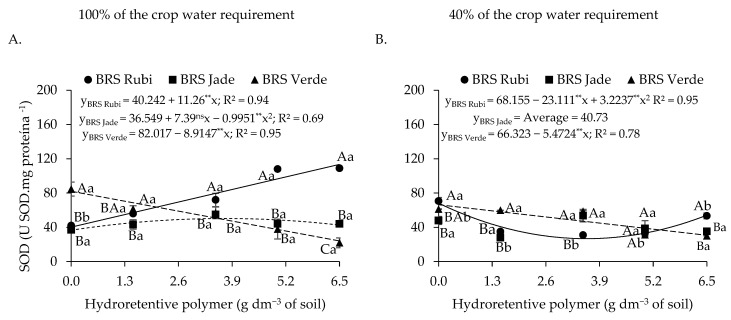
Superoxide dismutase activity—SOD (**A**,**B**) of cotton cultivars under irrigation levels (100 and 40% of the crop water requirement) and hydroretentive polymer application at 77 days after sowing in Experiment II. Identical uppercase letters indicate no significant differences among the different cultivars (Tukey test, *p* ≤ 0.05), whereas identical lowercase letters indicate no significant differences among hydrogel polymer doses (Scott–Knott test, *p* ≤ 0.05). ^ns^ and ** indicate not significant and significant at *p* ≤ 0.01, respectively. Vertical bars represent the standard error of the mean (*n* = 3).

Regarding superoxide dismutase (SOD) activity under full irrigation conditions (Figure 15A), cv. BRS Rubi exhibited an increase of 181.87% at the dose of 6.5 g dm^−3^ of soil compared with the control. In contrast, cv. BRS Verde showed a linear reduction of 70.65% in SOD activity. Cv. BRS Jade displayed a quadratic response, with an increase of 37.54% (13.72 U SOD mg protein^−1^) at the estimated dose of 3.7 g dm^−3^ of soil compared with the control; however, above this dose, a reduction of 15.36% was observed when compared with the highest evaluated dose. Under deficit irrigation (Figure 15B), residual doses reduced SOD activity in cv. BRS Rubi, with a reduction of 60.77% at the estimated dose of 3.6 g dm^−3^ of soil relative to the control. In cv. BRS Verde, a linear decrease of 8.25% per unit increase in polymer dose was observed, resulting in a reduction of 53.63% at the dose of 6.5 g dm^−3^ of soil compared with the 0 g dm^−3^ dose.

**Figure 16:**
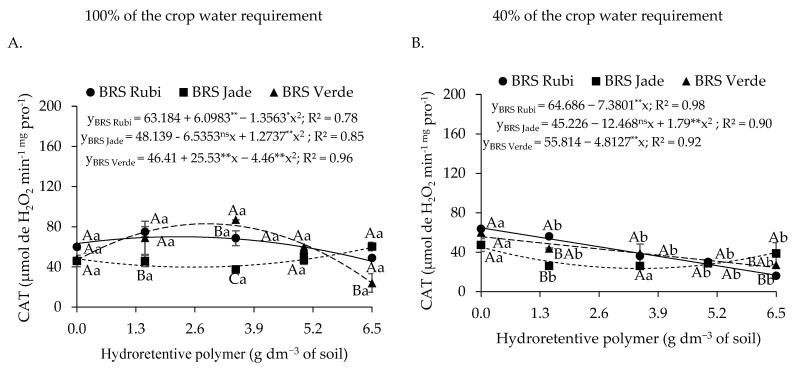
Catalase activity—CAT (**A**,**B**) of cotton cultivars under irrigation levels (100 and 40% of the crop water requirement) and hydroretentive polymer application at 77 days after sowing in Experiment II. Identical uppercase letters indicate no significant differences among the different cultivars (Tukey test, *p* ≤ 0.05), whereas identical lowercase letters indicate no significant differences among hydrogel polymer doses (Scott–Knott test, *p* ≤ 0.05). ^ns^, **, and * indicate not significant, significant at *p* ≤ 0.01, and significant at *p* ≤ 0.05, respectively. Vertical bars represent the standard error of the mean (*n* = 3).

In plants under full irrigation (Figure 15A), differences among cultivars were observed at all residual polymer doses. However, cv. BRS Rubi exhibited the highest SOD activity at doses of 3.5, 5.0, and 6.5 g dm^−3^ of soil, with increases of 26.32%, 184.21%, and 395.45%, respectively, compared with cv. BRS Verde. Under water deficit conditions (Figure 16B), differences were detected only at the lowest and highest hydroretentive polymer doses, with higher SOD activity observed in cv. BRS Rubi at doses of 0 and 6.5 g dm^−3^ of soil. Overall, higher SOD activity was observed in plants irrigated with 100% of the crop water requirement. However, under deficit irrigation in cv. BRS Verde, residual doses of 5.0 and 6.5 g dm^−3^ of the hydroretentive polymer resulted in increases of 4.05% and 27.72%, respectively, compared with plants of the same cultivar grown under the same conditions but irrigated with 100% of the crop water requirement. In contrast, cv. BRS Rubi exhibited the highest antioxidant activity under full irrigation at doses of 1.5, 3.5, 5.0, and 6.5 g dm^−3^ of soil, with values higher than those recorded under deficit irrigation.

In Figure 16A, plants under full irrigation exhibited a quadratic response to residual doses of the hydroretentive polymer for catalase (CAT) activity. Cultivars BRS Rubi and BRS Verde showed increases of 10.80% and 78.68%, respectively, when subjected to the estimated doses of 2.2 and 2.9 g dm^−3^ of soil, compared with the control. However, above these doses, reductions in CAT activity of 35.01% and 71.16% were observed at the dose of 6.5 g dm^−3^ of soil. A contrasting response was observed in cv. BRS Jade, which exhibited a reduction of 17.41% at the estimated dose of 2.6 g dm^−3^ of soil relative to the control. Under deficit irrigation conditions (Figure 16B), cvs. BRS Rubi and BRS Verde showed a linear response, with reductions in CAT activity of 11.41% and 8.62%, respectively, per unit increase in polymer dose. For cv. BRS Jade, a reduction of 48.03% was observed at the estimated dose of 3.5 g dm^−3^ of soil compared with the control; above this dose, an increase of 69.03% was recorded relative to the highest evaluated dose.

Under full irrigation, no significant differences among cultivars were observed at doses of 0 and 5.0 g dm^−3^ of soil, whereas CAT activity was higher in cv. BRS Verde at the dose of 3.5 g dm^−3^ of soil compared with the other cultivars. For plants grown under deficit irrigation, significant differences among cultivars were observed at doses of 1.5 and 6.5 g dm^−3^ of soil, with cvs. BRS Rubi and BRS Jade showing higher CAT activity at these respective doses. Overall, higher CAT activity was observed in plants irrigated with 100% of the crop water requirement (Figure 16A,B) in cvs. BRS Rubi and BRS Jade, with expressive increases of 29.21%, 74.78%, 115.10%, and 172.31% for cv. BRS Rubi, and 34.99%, 74.36%, 71.90%, and 50.00% for cv. BRS Jade at doses of 1.5, 3.5, 5.0, and 6.5 g dm^−3^ of soil of the hydroretentive polymer, respectively, compared with plants grown under the same doses but irrigated with 40% of the crop water requirement.

**Figure 17:**
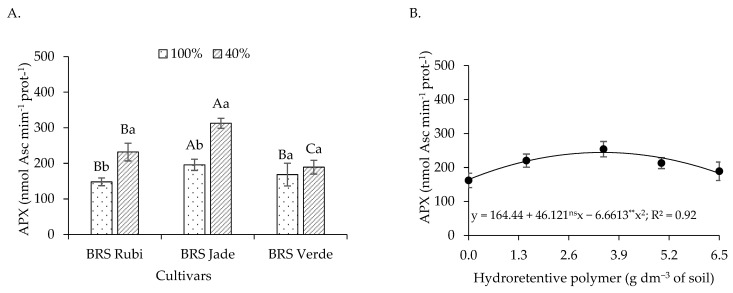
Ascorbate peroxidase activity—APX (**A**,**B**) of cotton cultivars under irrigation levels (100 and 40% of the crop water requirement) and hydroretentive polymer application at 77 days after sowing in Experiment II. Identical uppercase letters indicate no significant differences among the different cultivars (Tukey test, *p* ≤ 0.05), whereas identical lowercase letters indicate no significant differences among hydrogel polymer doses (Scott–Knott test, *p* ≤ 0.05). ^ns^ and ** indicate not significant and significant at *p* ≤ 0.01, respectively. Vertical bars represent the standard error of the mean (*n* = 3).

With respect to plants grown under full irrigation, cv. BRS Jade exhibited the highest ascorbate peroxidase (APX) activity, with a value of 196 nmol ASC min^−1^ mg protein^−1^, being 32.32% and 16.35% higher than those observed in cvs. BRS Rubi and BRS Verde, respectively. When irrigation levels were analyzed within each cultivar, deficit irrigation increased APX activity by 56.44% and 59.52% in cotton cvs. BRS Rubi and BRS Jade, respectively (Figure 17A). The highest estimated value of 312.66 nmol ASC min^−1^ mg protein^−1^ was recorded in cv. BRS Jade irrigated with 40% of the crop water requirement.

Ascorbate peroxidase activity was quadratically affected by hydroretentive polymer doses (Figure 17B), with the highest estimated value of 244.26 nmol ASC min^−1^ mg protein^−1^ observed at the estimated dose of 3.5 g dm^−3^ of soil. This value represented an increase of 48.54% (79.82 nmol ASC min^−1^ mg protein^−1^) compared with the control.

## 3. Discussion

In semiarid regions, particularly in the Brazilian Northeast semiarid zone, the irregularity and poor distribution of rainfall, combined with high temperatures, compromise water availability, constituting one of the main limiting factors for agricultural production [35]. Water stress promotes significant changes in plant physiological mechanisms, such as reductions in protein synthesis and enzymatic activity, resulting in metabolic imbalances that impair crop growth, development, and productivity [36].

Thus, improving water use efficiency and increasing agricultural productivity are among the major challenges facing modern agriculture [37]. In this context, polymer-based materials with high capacity for absorbing and retaining irrigation water have emerged as promising alternatives for water management in agricultural systems [38]. Hydrogels, or hydroretentive polymers, act as soil microreservoirs, retaining water during irrigation and gradually releasing it as soil moisture decreases, thereby enhancing water availability to plants [33].

Under full irrigation conditions, plants generally exhibit adequate water status [39], as also observed in Experiments I and II. However, a reduction in this variable was observed as a function of hydroretentive polymer application. This response may be associated with changes in the saturated hydraulic conductivity of the soil, as well as with water diffusion processes [40]. Such limitations in soil water movement may have compromised water availability to plants, resulting in reductions in relative water content.

When plants are subjected to water deficit, a reduction in cellular water content is commonly observed, as this status reflects the degree of tissue hydration, particularly the plant’s capacity to retain water when exposed to abiotic stress [41]. However, this effect was mitigated by hydroretentive polymer application in both experiments for cvs. BRS Rubi and BRS Verde. These results are consistent with the findings of M’barki et al. [42], who reported higher relative water content values in olive (*Olea europaea* L.) seedlings subjected to rainfed conditions and treated with 3 g L^−1^ of hydroretentive polymer, showing superiority compared with plants grown under the same water conditions without polymer application.

As observed, plants subjected to water stress tend to exhibit reduced water content in their tissues, which renders cells more flaccid and may favor ion leakage, particularly when changes in turgor pressure occur, resulting in electrolyte leakage. In Experiment I, deficit irrigation negatively affected cell membrane integrity, as indicated by the increase in electrolyte leakage (EL) in cvs. BRS Rubi and BRS Verde (Figure 3A). This increase in EL under water deficit conditions indicates greater structural disorganization of membranes, which is often associated with excessive accumulation of reactive oxygen species (ROS), leading to oxidative damage to membrane lipids [43].

However, reductions in electrolyte leakage were observed as hydroretentive polymer doses increased. This finding suggests that hydrogel application to the soil improved water retention and maintained soil moisture near the root zone (Figure 3B), which favors greater cellular turgor and prevents electrolyte leakage. Similar results were reported by Farahani et al. [32] when evaluating hydrogel application in lemon balm (*Melissa officinalis* L.) plants under water deficit conditions, in which the application of 1 g kg^−1^ of soil of a hydroretentive polymer reduced electrolyte leakage. In addition, the data obtained in Experiment II reinforce the effectiveness of hydrogel application for this variable, even under residual effect conditions, as electrolyte leakage was reduced in plants subjected to deficit irrigation (Figure 11B).

Reductions in gas exchange variables were observed under deficit irrigation. These responses may also be associated with lower water availability, which leads to reduced cellular turgor, since partial stomatal closure represents a physiological mechanism used by plants to minimize water loss through transpiration to the atmosphere, thereby contributing to the conservation of leaf water potential [44]. However, this strategy also restricts CO_2_ diffusion into the leaf, which may limit the CO_2_ assimilation rate [45].

In Experiments I and II, an increase in stomatal conductance was observed as a function of hydroretentive polymer doses and their residual effects, respectively, under full and deficit irrigation conditions. However, the increase in *gs* also intensified water vapor loss through transpiration, thereby favoring water loss to the atmosphere. These results are consistent with the findings of Souto et al. [46], who reported increased stomatal conductance in sour passion fruit plants under full irrigation at a dose of 1.06 g dm^−3^ of soil, as well as superior performance of plants under deficit irrigation at a hydrogel dose of 1.0 g dm^−3^.

In Experiment I, an increase in leaf transpiration (*E*) was observed in cotton plants subjected to both full irrigation (Figure 5A) and deficit irrigation (Figure 5B), except for cv. BRS Rubi. In Experiment II, the residual dose of 3.3 g dm^−3^ of soil also increased E under water restriction conditions. These results differ from those reported by Silvério et al. [47], who observed a reduction in transpiration in seedlings of *Eugenia myrcianthes* Nied. under water deficit when a hydroretentive polymer was applied. Water restriction generally negatively affects transpiration; however, it also represents an adaptive strategy to minimize excessive water loss. Nevertheless, this physiological mechanism limits CO_2_ entry, which is essential for the Calvin cycle and, consequently, for photosynthesis [48].

Regarding the CO_2_ assimilation rate (*A*), in Experiments I and II the highest values were observed in plants subjected to deficit irrigation (Table 1; Figure 14). This effect may be attributed to improved maintenance of soil moisture near the plant root system, which enhances water availability and consequently contributes to increases in the CO_2_ assimilation rate [46,49].

In general, water stress reduces the contents of chlorophyll *a*, chlorophyll *b*, total chlorophyll, and carotenoids, thereby impairing photosynthetic efficiency [19]. However, an opposite effect was observed in the present study, in which increases in these pigment contents were detected under deficit irrigation. Corroborating this finding, a study conducted by González−Espíndola et al. [50] on birdsfoot trefoil (*Lotus corniculatus* L.) reported higher concentrations of photosynthetic pigments under water deficit conditions, which may indicate a potential adaptive response aimed at maintaining photosynthetic efficiency.

In Experiment I, increases in photosynthetic pigment contents (chlorophyll *a*, chlorophyll *b*, total chlorophyll, and carotenoids) were observed in plants grown under water restriction and hydrogel application. In Experiment II, increases in these variables were recorded in plants under full irrigation and the residual effect of the hydroretentive polymer. These results are consistent with those reported by Ghobashy et al. [51], who observed increased contents of photosynthetic pigments in *Pisum sativum* plants under both full irrigation and water stress conditions following hydroretentive polymer application. Under similar conditions, Liu et al. [22] and Ramadan et al. [19] also reported increases in photosynthetic pigment contents in spinach (*Spinacia oleracea*) and faba bean (*Vicia faba*), respectively. Increased chlorophyll contents are associated with a greater capacity of plants to perform photosynthesis [22], mainly due to higher efficiency during the photochemical phase.

Under deficit irrigation, an increase in initial fluorescence (F_o_) was observed, which may indicate inefficiency in the light-harvesting system of photosystem II (PSII), thereby increasing energy dissipation through fluorescence [52,53]. In addition, reductions in maximum fluorescence, variable fluorescence, and the maximum quantum efficiency of PSII directly reflect a decrease in the maximum photochemical efficiency of PSII, indicating that most PSII reaction centers are not functional in the conversion of light energy into chemical energy [18,54].

Conversely, in Experiment I, reductions in initial fluorescence (F_o_) were observed in plants under full irrigation, except for cv. BRS Verde (Figure 6A), as well as in plants subjected to deficit irrigation (Figure 6B). The beneficial effect of the residual polymer dose on F_0_ in Experiment II for cv. BRS Jade under water restriction may be associated with improved photochemical efficiency. Similarly, Araújo et al. [55], working with yellow passion fruit cultivated under deficit irrigation conditions, reported a reduction in F_o_ following the application of a polymer dose of 2.0 g dm^−3^ in a semiarid region.

In both experiments, the hydroretentive polymer increased maximum fluorescence (Fm), variable fluorescence (Fv), and the maximum quantum efficiency of photosystem II (Fv/Fm) in plants under deficit irrigation conditions. Similar results were reported by Santos et al. [56] in seedlings of Brazilian pepper tree (*Schinus terebinthifolia* Raddi) subjected to water deficit and hydrogel application, who observed increases in Fv/Fm. Araújo et al. [55], who studied the functional integrity of the photosynthetic apparatus and optimized plant photosynthetic performance, also reported increases in Fm and Fv in leaves of yellow passion fruit cultivated with a hydroretentive polymer at a dose of 1.0 g dm^−3^ of soil. These responses may be associated with reduced degradation of the D1 protein, an essential component of the PSII occurrence center, and for mitigating oxidative damage, thus supporting the functional integrity of the photosynthetic apparatus and optimizing the photosynthetic performance of plants [57].

The reduction in total soluble protein (TSP) contents in plants subjected to water restriction may be associated with oxidative damage caused by excessive accumulation of reactive oxygen species (ROS) [11]. In both experiments, increases in TSP levels were observed in plants under deficit irrigation when hydroretentive polymer doses were applied. The results obtained in this study are consistent with those reported by Fidelis et al. [58] in cowpea (*Vigna unguiculata* L.) and by Ramadan et al. [19] in faba bean. The increase in TSP contents may be attributed to greater moisture retention in the root zone promoted by the hydrogel, which contributed to the maintenance of metabolic functions such as protein synthesis.

In addition, this increase may be related to the activation of stress-associated proteins, such as late embryogenesis abundant (LEA) proteins, which act as protective molecules under stress conditions and can enhance plant tolerance [59]. Furthermore, plants may induce the synthesis of new proteins that are not present under normal conditions, leading to changes in gene expression and thereby contributing to cell membrane stability [60].

Excessive production of reactive oxygen species (ROS) under water deficit conditions induces oxidative stress at the cellular level, including lipid peroxidation, protein oxidation, and damage to nucleic acids [61]. To counteract the deleterious effects of ROS, plants possess an enzymatic antioxidant defense system that includes superoxide dismutase (SOD), ascorbate peroxidase (APX), and catalase (CAT), which act to mitigate oxidative stress [62].

Superoxide dismutase (SOD) constitutes the first line of enzymatic antioxidant defense in plant cells, playing an essential role in the elimination of reactive oxygen species (ROS). Its primary function is to catalyze the dismutation of the superoxide anion (O_2_•^−^) into molecular oxygen and hydrogen peroxide, using metal cofactors [63]. In Experiment I, lower SOD activity was observed in cv. BRS Jade under deficit irrigation and hydrogel application. In Experiment II, however, the cultivars exhibited distinct responses regarding SOD activity. Residual doses of the hydroretentive polymer induced SOD activation in plants under full irrigation, except in cv. BRS Verde, whereas under water restriction SOD activity was reduced (Figure 15A,B). The incorporation of hydroretentive polymer into the soil may significantly reduce the activity of antioxidant enzymes, possibly due to increased water retention capacity. The water-holding properties of the polymer favor the mitigation of oxidative damage associated with water stress, thereby contributing to the maintenance of plant homeostasis [64].

Catalase (CAT) acts in the detoxification of reactive oxygen species (ROS) produced mainly during cellular respiration and photosynthesis. Its primary function is to catalyze the decomposition of hydrogen peroxide (H_2_O_2_) into water (H_2_O) and molecular oxygen (O_2_), being capable of decomposing millions of H_2_O_2_ molecules per second [65]. In the present study, increases in CAT activity were observed in plants under full irrigation as a function of hydroretentive polymer doses (Figure 7 and Figure 16A). Conversely, plants subjected to deficit irrigation exhibited a reduction in CAT activity. In agreement with these findings, Gomaa and Aldaby [66] also reported reduced CAT activity in sunflower (*Helianthus annuus* L.) seedlings subjected to water deficit and hydroretentive polymer application. According to the authors, the decrease in CAT activity is associated with the physical conditioning of the soil promoted by the hydroretentive polymer.

Ascorbate peroxidase (APX) catalyzes the conversion of hydrogen peroxide (H_2_O_2_) into water (H_2_O), using ascorbate as an electron donor. Its expression is differentially regulated in response to environmental stresses and during plant growth and development under normal conditions [67]. In Experiments I and II, APX activity increased in plants grown under both full and deficit irrigation following hydroretentive polymer application to the soil. In a study with the medicinal plant *Satureja rechingeri* Jamzad, Beiranvandi et al. [68] also reported increased APX activity in plants subjected to water restriction and hydroretentive polymer doses.

The increase in the activity of SOD, CAT, and APX enzymes in plants grown under full irrigation and hydroretentive polymer doses may be associated with improved soil water conditioning [69], which favors higher photosynthetic efficiency. In turn, the intensification of the photosynthetic process results in increased production of reactive oxygen species (ROS), which act as signaling molecules in several plant growth and development processes [67,70]. In addition, the literature reports an increase in antioxidant activity in plants even under normal irrigation conditions, resulting from the use or absence of stress-attenuating agents [19,71].

## 4. Materials and Methods

**Figure 18:**
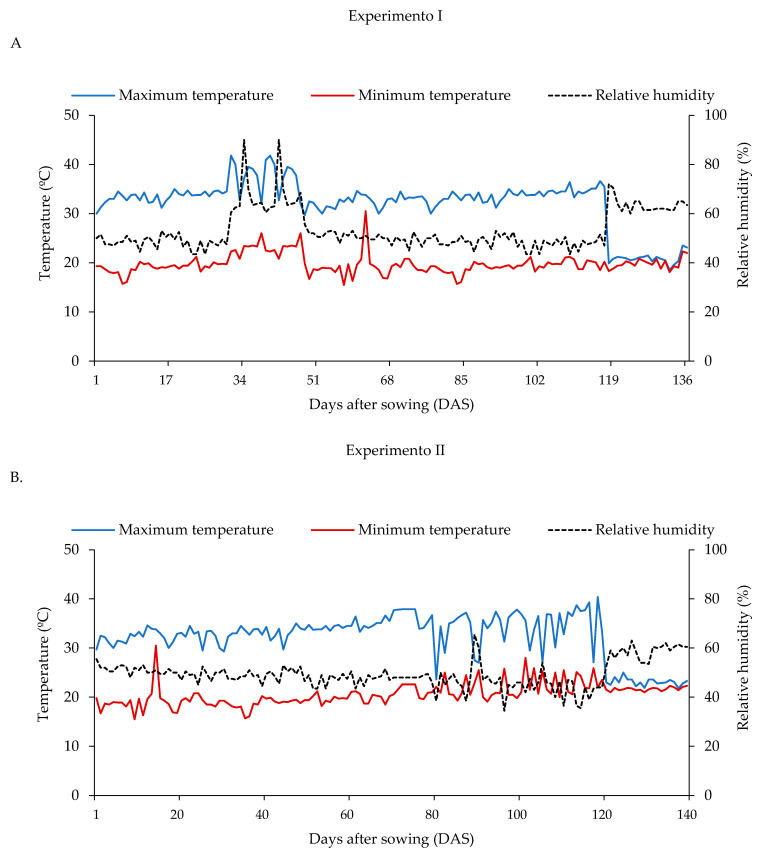
Maximum and minimum air temperatures and relative humidity during the experimental period from 1 to 14 July 2024 for Experiment I (**A**) and from 29 July to 15 December 2024 for Experiment II (**B**).

Two consecutive experiments were conducted from March to July 2024 and from July to December 2024 in a greenhouse at the Center for Technology and Natural Resources (CTRN), Federal University of Campina Grande (UFCG), located in the municipality of Campina Grande, Paraíba State, Brazil, at the geographical coordinates 07°15′18″ S latitude, 35°52′28″ W longitude, and an average altitude of 550 m. The regional climate is classified as As (tropical with winter rainfall and dry summer), according to the Köppen classification [72]. Air temperature and relative humidity data recorded inside the greenhouse were obtained using a digital thermos hygrometer (Pro Hygro Thermo, Garden HighPro, Jundiaí, Brazil) and are presented in Figure 18.

A randomized complete block design was used in both experiments, arranged in a 2 × 5 × 3 factorial scheme, consisting of two irrigation levels (L: 100 and 40% of the crop water requirement), five hydroretentive polymer doses (H: 0.0, 1.5, 3.5, 5.0, and 6.5 g dm^−3^ of soil), and three naturally colored cotton cultivars [C: BRS Rubi, BRS Jade, and BRS Verde, where BRS (Brazil Seeds)], with three replicates, totaling ninety experimental units (Table 7). In the second experiment, the residual effect of the hydroretentive polymer applied during the first experiment was evaluated. The hydroretentive polymer doses were based on a study conducted with yellow passion fruit (*Passiflora edulis* L.) [55], while the irrigation levels were defined according to a study on cotton [73].

Seeds of the cotton cultivars BRS Rubi, BRS Jade, and BRS Verde were used. Cultivar BRS Rubi has reddish-brown fibers, an average plant height of 110 cm, fiber length of 25.4 UHM (Ultra-High Modulus), fiber strength of 24.5 gf tex^−1^, and an average crop cycle of 140–150 days. Cultivar BRS Jade presents light-brown fibers, an average plant height of 120 cm, fiber length of 28.6 UHM, fiber strength of 29.2 gf tex^−1^, and an average crop cycle of 120–150 days. Cultivar BRS Verde is characterized by light green fibers, an average plant height of 127 cm, fiber length of 29.56 UHM, fiber strength of 25.86 gf tex^−1^, and an average crop cycle of 130–140 days.

Plants were grown in plastic containers (pots) with a capacity of 20 L (35 cm height × 31 cm upper diameter × 20 cm lower diameter). At the base of each lysimeter, a 15 mm diameter hose was installed to function as a drain and connected to a 2 L plastic container for collecting drained water. A polypropylene mesh was placed over the drain, followed by a 3 cm layer of gravel. Subsequently, the pots were filled up to half of their capacity with soil classified as a Regolithic Neosol with sandy loam texture, collected from the 0–30 cm depth. The physical hydric and chemical attributes of the soil were determined in the laboratory prior to treatment application [74]. The pots were arranged in single rows, spaced 1.5 m between rows and 1.0 m between plants within the row.

Before treatment application, the soil exhibited a water pH of 5.40, indicating a slightly acidic condition. Organic matter content was 17.62 g dm^−3^, while available phosphorus content was 2.92 mg dm^−3^. Regarding exchangeable cations, potassium (K^+^) and sodium (Na^+^) contents were 0.28 and 0.04 cmolc kg^−1^, respectively. Calcium (Ca^2+^) and magnesium (Mg^2+^) contents were 1.87 and 1.70 cmolc kg^−1^, respectively, whereas exchangeable aluminum (Al^3+^) presented a value of 0.20 cmolc kg^−1^, and hydrogen (H^+^) content was 0.85 cmolc kg^−1^. Concerning chemical attributes related to salinity and sodicity, the electrical conductivity of the saturation extract (ECw) was 0.72 dS m^−1^, indicating low salinity. Cation exchange capacity (CEC) was 6.94 cmolc kg^−1^, while the sodium adsorption ratio of the saturation extract (SAR_e_) was 0.03, reflecting a low risk of sodification. Exchangeable sodium percentage (ESP) was 0.58%, a value considered low and suitable for crop development. Regarding physical hydric attributes, particle-size analysis revealed the predominance of the sand fraction (675.2 g kg^−1^), followed by silt (221.1 g kg^−1^) and clay (103.7 g kg^−1^), classifying the soil as sandy to sandy loam in texture. The soil moisture at 0.3 kPa was 5.32 dag kg^−1^, while the moisture at 1500 kPa was 7.66 dag kg^−1^.

To meet the nutritional requirements of the plants, nitrogen, phosphorus, and potassium fertilization was initiated 30 days after sowing (DAS) and subsequently applied at 20-day intervals, following the recommendations of Novais et al. [75]. The equivalent of 100, 300, and 150 mg kg^−1^ of soil of N, P_2_O_5_, and K_2_O, respectively, was applied via fertigation, divided into three applications, using urea (45% N), monoammonium phosphate—MAP (60% P_2_O_5_ and 12% N), and potassium chloride (60% K_2_O). Micronutrient fertilization was performed via foliar application starting at 30 DAS, at 20-day intervals. Applications were carried out on both the adaxial and abaxial leaf surfaces using a micronutrient compound (Dripsol© Micro) containing Mg^2+^ (1.1%), B (0.85%), Cu (Cu−EDTA) (0.5%), Fe (Fe−EDTA) (3.4%), Mn (Mn−EDTA) (3.2%), Mo (0.05%), and Zn (4.2%), with 70% chelating agent (EDTA), applied at a concentration of 1 g L^−1^.

Prior to sowing, the hydroretentive polymer Forth^®^ (Forth, Cerquilho, Brazil) was incorporated into the soil according to the treatments (1.5, 3.5, 5.0, and 6.5 g dm^−3^ of soil). After its incorporation, a soil layer was added over the polymer. The polymer consists of a potassium polyacrylate polyacrylamide copolymer, with a cation exchange capacity (CEC) of 532.26 mmolc dm^−3^ and a water retention capacity (WRC) of 1526.69%. The conditioner is classified as Class E, granular type, designed for soil application, and consists of a synthetic or mineral material with predominantly physical action, acting in soil water retention. The hydrogel was diluted to 4 g L^−1^ and hydrated for 24 h prior to application, following the manufacturer’s recommendations. The control treatment did not receive polymer addition to the substrate.

Five seeds were sown per lysimeter at a depth of 2 cm and evenly distributed. After sowing, soil moisture was maintained close to field capacity until the emergence of the third fully expanded leaf (19 and 21 days after sowing DAS for Experiments I and II, respectively), when treatment application began. During this period, thinning was performed, leaving one plant per lysimeter, and treatment differentiation was initiated.

Prior to sowing, the volume of water required for the soil to reach field capacity was determined. After sowing, irrigation was performed daily at 17:00 h, applying the water volume corresponding to each irrigation level (100 and 40% of the crop water requirement), based on the soil water balance in the root zone.

Phytosanitary control of pests and diseases was carried out through preventive interventions using synthetic chemical insecticides, acaricides, and fungicides, with the active ingredients chlorfenapyr, mancozeb, and imidacloprid, applied as needed for the control of cotton bollworm (*Heliothis virescens*), broad mite (*Polyphagotarsonemus latus*), anthracnose (*Colletotrichum truncatum*), aphid (*Aphis gossypii*), and whitefly (*Bemisia tabaci*). Applications were performed using a manual pre-compression sprayer with a volumetric capacity of 20 L. Additionally, superficial soil scarification was carried out to improve soil aeration, along with manual removal of weeds.

At 75 and 77 days after sowing (DAS), for Experiments I and II, respectively, the following variables were evaluated: relative water content (RWC), electrolyte leakage (EL), chlorophyll *a* fluorescence, photosynthetic pigment contents, total soluble protein content, and antioxidant enzyme activity.

To determine relative water content (RWC), one leaf was collected from the middle third of the productive branch, from which ten discs with a diameter of 1.54 cm^2^ were obtained. For RWC determination, five discs were immediately weighed to obtain fresh mass (FM), according to the methodology described by Weatherley [76]. Electrolyte leakage (EL) was determined using the five leaf discs and expressed as a percentage, according to the methodology proposed by Scotti-Campos et al. [77].

Gas exchange measurements were performed between 7:00 and 9:00 a.m., quantifying stomatal conductance (*gs*), transpiration (*E*), intercellular CO_2_ concentration (*Ci*), and CO_2_ assimilation rate (*A*). Measurements were carried out using an infrared gas analyzer—IRGA (Infra Red Gas Analyser, model LCpro-SD, ADC Bioscientific, Hoddesdon, UK). Readings were obtained from the third fully expanded leaf, counted from the apical bud, under natural air temperature and ambient CO_2_ concentration, using an artificial light source providing a photosynthetic photon flux density of 1200 μmol m^−2^ s^−1^.

Photosynthetic pigment contents were determined according to the methodology adapted from Arnon [78], in which chlorophyll *a*, chlorophyll *b*, total chlorophyll, and carotenoid contents in the leaves were expressed in μg mL^−1^.To determine initial fluorescence (F_0_), maximum fluorescence (Fm), variable fluorescence (Fv), and maximum quantum efficiency of photosystem II (Fv/Fm), leaf clips were attached, and, after a 30 min dark adaptation period, measurements were performed using a pulse-amplitude modulation fluorometer (PAM fluorometer, model OS5p, Opti-Sciences, Hudson, NY, USA).

For the quantification of total soluble protein content and antioxidant enzyme activity, the fourth fully expanded leaf, counted from the apex to the base, was collected from each treatment at 5:00 a.m., following the method described by Dutra et al. [79]. Total soluble protein (TSP) content was determined according to the method described by Bradford [80]. Superoxide dismutase (SOD; EC 1.15.1.1) activity was determined based on the enzyme’s ability to inhibit the photoreduction of nitroblue tetrazolium chloride (NBT), using a method adapted from Beauchamp and Fridovich [81]. The results were expressed as U SOD mg^−1^ protein [82].

Catalase (CAT; EC 1.11.1.6) activity was quantified according to Sudhakar et al. [83], based on the consumption of hydrogen peroxide (H_2_O_2_) by the enzyme present in the extract, with enzymatic activity calculated using the Lambert–Beer method [84]. Ascorbate peroxidase (APX; EC 1.11.11.1) activity was determined based on ascorbate consumption, following the method of Nakano and Asada [85]. Final APX activity was expressed as nmol ascorbate min mg^−1^ protein. It is important to emphasize that TSP, SOD, CAT, and APX determinations were performed in triplicate.

The experimental data were subjected to preliminary procedures to verify statistical assumptions. Initially, the observed values for each variable were organized according to the experimental treatments and examined for outliers and inconsistencies. Subsequently, residual normality was assessed using the Shapiro–Wilk test, while homogeneity of variances (homoscedasticity) among treatments was verified using Levene’s test.

After these procedures, the data were subjected to multivariate analysis using Principal Component Analysis (PCA), which allowed dimensionality reduction in the original dataset while preserving the most relevant information through linear combinations of variables. Component selection was based on eigenvalues (λ ≥ 1.0) from the correlation matrix, provided that they explained more than 10% of the total variance [86].

After this step, the variables grouped within each component were evaluated using multivariate analysis of variance (MANOVA), applying Hotelling’s test at a 5% significance level for the factors irrigation levels, cotton cultivars, hydrogel polymer doses, and their interactions. Only variables with correlation coefficients ≥ 0.60 within each principal component were retained. Subsequently, orthogonal Varimax rotation was applied to facilitate interpretation of factor loadings while maintaining orthogonality among components. All statistical analyses were performed using RStudio (version 4.1.0), with the support of the packages ggcorrplot, mclust, psych, ggplot2, ggrepel, and factoextra.

Variables with correlation coefficients lower than 0.60 were subjected to analysis of variance (ANOVA) using the F test (*p* ≤ 0.05). When significant, polynomial regression analysis (linear and quadratic) was performed for hydrogel polymer doses. The Scott–Knott test (*p* ≤ 0.05) and Tukey test (*p* ≤ 0.05) were used for mean comparisons among irrigation levels and cultivars, respectively, using the SISVAR ESAL software, version 5.8 [87].

## 5. Conclusions

Water deficit negatively affected relative water content, electrolyte leakage, gas exchange, chlorophyll *a* fluorescence, and antioxidant activity in cotton cultivars. However, more pronounced damage was observed in cv. BRS Rubi. The application of the hydroretentive polymer at concentrations up to 6.5 g dm^−3^ of soil promoted positive effects on the physiology and antioxidant mechanisms of naturally colored cotton under water-restricted conditions. The use of residual polymer doses increased relative water content and antioxidant activity, improved gas exchange and chlorophyll fluorescence, and contributed to the maintenance of plant physiological health in both experiments. In addition, polymer application, both during the initial cultivation cycle and as a residual effect in the subsequent cycle, proved to be an efficient strategy to mitigate the effects of water stress by reducing cell membrane permeability and the activity of enzymes associated with oxidative stress. This response may favor the development of the cultivars BRS Rubi, BRS Jade, and BRS Verde under water deficit conditions. Overall, the results indicate that the use of hydroretentive polymers represents a promising alternative for agriculture in semiarid regions, enhancing the resilience of naturally colored cotton to water deficit and contributing to the sustainability of the cropping system. Nevertheless, future studies should explore cultivar variability and optimize application rates to maximize the benefits of this technology, as well as validate these findings under field conditions.

## Figures and Tables

**Table 1:** Eigenvalues and percentage of total variance explained in the multivariate analysis of variance, with significance probability based on Hotelling’s test (*p*-value) for irrigation levels, hydroretentive polymer doses, and cotton cultivars in Experiment I.

								Main Components			
								PC_1_		PC_2_	
Eigenvalues (ʎ)								5.73		2.25	
Percentage of total variance (S^2^%)								52.13		20.46	
Hotteling test for applied blades (L)								0.01 **		0.05 *	
Hotteling test for cultivars (C)								0.01 **		0.01 **	
Hotteling test for hydroretentive polymer (H)								0.01 **		0.01 **	
Hotteling interaction test (L × C)								0.01 **		0.01 **	
Hotteling test for the interaction (L × H)								0.01 **		0.01 **	
Hotteling test for the interaction (C × H)								0.01 **		0.01 **	
Hotteling test for the interaction (L × C × H)								0.01 **		0.01 **	
PCs	Correlation coefficients (r)										
	Chl *a*	Chl *b*	Chl t	Car	*Ci*	*A*	Fm	Fv	Fv/Fm	TSP	SOD
PC_1_	0.92 ***	0.92 ***	0.94 ***	0.76 ***	−0.77 **	0.07 ^ns^	−0.21 ^ns^	−0.20 ^ns^	0.84 ***	0.89 ***	−0.64 ***
PC_2_	−0.04 ^ns^	−0.21 ^ns^	−0.09 ^ns^	−0.20 ^ns^	0.12 ^ns^	0.64 ***	0.90 ***	0.93 ***	−0.01 ^ns^	−0.11 ^ns^	0.22 ^ns^

*,**, ***, ^ns^—Significant at *p* ≤ 0.05, *p* ≤ 0.01, *p* ≤ 0.001, and not significant, respectively.

**Table 2:** Mean values of chlorophyll *a* (Chl *a*; μg mL^−1^), chlorophyll *b* (Chl *b*; μg mL^−1^), total chlorophyll (Chl *t*; μg mL^−1^), carotenoids (Car; μg mL^−1^), intercellular CO_2_ concentration (*Ci*; μmol CO_2_ m^−2^ s^−1^), CO_2_ assimilation rate (*A*; μmol CO_2_ m^−2^ s^−1^), maximum fluorescence (Fm), variable fluorescence (Fv), maximum quantum efficiency of photosystem II (Fv/Fm), total soluble proteins (TSP; mg mL^−1^), and superoxide dismutase activity (SOD; U SOD mg protein^−1^) as a function of irrigation levels, hydroretentive polymer doses, and cotton cultivars at 75 days after sowing in Experiment I.

Treatments	Chl *a*	Chl *b*	Chl t	Car	*Ci*	*A*	Fm	Fv	Fv/Fm	TSP	SOD
L_1_C_1_H_1_	1137.23d ± 31.40	475.84e ± 5.81	1613.07e ± 16.70	213.21e ± 14.58	239.33b ± 5.49	15.66d ± 0.24	1172.33b ± 6.07	922.33b ± 5.61	0.787b ± 0.002	0.991d ± 0.009	75.37b ± 1.66
L_1_C_1_H_2_	1030.80d ± 44.53	504.12e ± 4.83	1534.92e ± 22.27	250.01d ± 5.97	239.67b ± 2.03	17.27c ± 0.68	1228.00a ± 21.38	980.00a ± 7.51	0.780c ± 0.004	1.057d ± 0.132	68.33c ± 0.20
L_1_C_1_H_3_	931.09e ± 1.52	521.09e ± 15.68	1452.18e ± 17.22	273.24c ± 22.64	246.67b ± 6.09	15.61d ± 0.52	1149.00b ± 14.35	907.00b ± 37.86	0.784c ± 0.006	1.173d ± 0.128	62.09d ± 1.50
L_1_C_1_H_4_	923.39e ± 16.82	561.18d ± 3.28	1484.57e ± 12.16	265.29c ± 6.87	249.00b ± 13.67	14.93d ± 0.28	1099.33c ± 49.45	862.33c ± 8.22	0.780c ± 0.008	1.236d ± 0.094	53.01e ± 0.76
L_1_C_1_H_5_	912.77e ± 10.85	660.45c ± 14.15	1573.22e ± 20.80	224.43e ± 25.15	255.33a ± 13.00	14.37e ± 0.27	1093.33c ± 33.61	863.33c ± 12.62	0.784c ± 0.003	1.463c ± 0.172	70.20c ± 8.11
L_1_C_2_H_1_	1078.15d ± 0.01	619.01c ± 22.40	1697.16e ± 35.94	273.07c ± 6.46	244.33b ± 5.61	18.85b ± 0.27	1068.00d ± 9.82	854.00c ± 9.82	0.790b ± 0.006	1.013d ± 0.056	81.53a ± 3.18
L_1_C_2_H_2_	1132.44d ± 1.10	557.21d ± 18.49	1689.65e ± 11.52	250.92d ± 6.53	249.67b ± 5.90	18.84b ± 0.44	1105.33c ± 14.16	954.33a ± 14.16	0.782c ± 0.005	1.092d ± 0.158	76.28b ± 1.63
L_1_C_2_H_3_	1086.48d ± 0.62	479.33e ± 21.17	1565.81e ± 32.02	240.73d ± 1.31	269.00a ± 5.12	19.03b ± 0.10	1128.00b ± 0.10	933.00b ± 2.00	0.783c ± 0.005	1.140d ± 0.040	61.69d ±3.71
L_1_C_2_H_4_	1374.97c ± 6.14	523.07e ± 7.37	1898.04d ± 16.10	238.20d ± 0.99	244.67b ± 4.33	18.08c ± 0.12	1148.33b ± 2.67	941.33b ± 6.12	0.783c ± 0.002	1.078d ± 0.046	53.41e ± 3.18
L_1_C_2_H_5_	1476.35c ± 47.20	669.16c ± 15.94	2145.50c ± 26.68	223.14e ± 1.90	238.33b ± 5.24	16.87c ± 0.11	1183.33a ± 34.97	948.33a ± 5.68	0.783c ± 0.004	1.038d ± 0.052	46.05f ± 1.66
L_1_C_3_H_1_	1074.47d ± 44.57	505.03e ± 9.29	1579.50e ± 18.79	256.15c ± 3.71	280.00a ± 1.56	14.05e ± 0.37	1170.67b ± 6.31	941.67b ± 29.01	0.782c ± 0.005	0.925d ± 0.010	84.41a ± 3.72
L_1_C_3_H_2_	1147.57d ± 19.68	559.15d ± 22.68	1706.72e ± 25.90	251.39d ± 9.97	256.00a ± 2.31	18.05c ± 0.62	1141.67b ± 11.60	890.67c ± 42.86	0.778b ± 0.008	1.057d ± 0.058	73.86b ± 2.67
L_1_C_3_H_3_	1204.74d ± 27.90	590.55c ± 1.64	1795.29d ± 16.78	246.16d ± 18.16	244.00b ± 6.35	15.88d ± 0.10	1136.67b ± 13.44	880.33c ± 15.91	0.778c ± 0.008	1.090d ± 0.025	52.63e ±2.15
L_1_C_3_H_4_	998.60e ± 64.38	616.93c ± 12.80	1615.52e ± 25.92	244.15d ± 3.04	242.67b ± 4.26	15.21d ± 0.27	1110.33c ± 23.41	854.33c ± 23.12	0.782b ± 0.002	1.147d ± 0.037	36.31g ± 2.11
L_1_C_3_H_5_	964.21e ± 25.52	627.25c ± 29.72	1591.46e ± 3.89	267.88c ± 2.66	232.00c ± 3.00	14.96d ± 0.02	1100.67c ± 1.35	833.67c ± 11.58	0.780b ± 0.001	1.201d ± 0.001	29.41h ± 2.17
L_2_C_1_H_1_	1446.82c ± 0.32	778.21b ± 3.59	2225.03c ± 47.59	257.62c ± 16.34	220.00c ± 7.51	11.84f ± 0.65	1068.00d ± 0.12	810.00d ± 11.08	0.801b ± 0.009	1.740b ± 0.059	27.92h ± 1.01
L_2_C_1_H_2_	1472.06c ± 3.70	789.81b ± 47.85	2261.87c ± 16.37	312.60b ± 3.95	213.00c ± 5.21	18.13c ± 0.48	1126.67b ± 15.34	884.67c ± 00.45	0.793b ± 0.003	1.840b ± 0.083	30.83h ± 1.36
L_2_C_1_H_3_	1618.45b ± 13.77	795.58b ± 36.98	2414.03c ± 25.22	329.47b ± 6.48	211.00c ± 2.52	16.97c ± 0.20	1131.33b ± 30.06	901.00b ± 25.05	0.798b ± 0.003	1.811b ± 0.030	35.05g ± 1.18
L_2_C_1_H_4_	1708.05b ± 38.35	860.74a ± 52.91	2568.79b ± 3.27	311.51b ± 13.47	213.00c ± 10.98	13.91e ± 0.36	1103.00c ± 17.34	875.67c ± 11.85	0.798b ± 0.007	1.665b ± 0.259	36.45g ± 1.81
L_2_C_1_H_5_	1744.34b ± 34.15	890.47a ± 18.67	2634.81b ± 47.86	247.66d ± 10.41	223.67b ± 4.17	11.33f ± 0.36	1044.00d ± 16.56	796.00d ± 41.56	0.794b ± 0.003	1.500c ± 0.233	78.15b ± 1.83
L_2_C_2_H_1_	1536.17c ± 58.19	720.43b ± 11.74	2256.60c ± 3.60	281.56c ± 11.80	186.33d ± 0.83	13.01f ± 0.09	1055.00d ± 0.15	812.67d ± 0.66	0.806a ± 0.006	1.927a ± 0.137	29.85h ± 1.81
L_2_C_2_H_2_	1807.03b ±6.84	947.48a ± 26.45	2754.51a ± 50.20	328.93b ± 7.61	208.67c ± 0.33	18.74b ± 0.61	1164.33b ± 7.22	942.33a ± 6.49	0.803b ± 0.022	2.038a ± 0.129	45.26f ± 1.83
L_2_C_2_H_3_	1978.81a ± 13.85	938.76a ± 16.02	2917.57a ± 43.15	347.14b ± 6.48	216.00d ± 8.09	22.39a ± 0.82	1096.67c ± 25.48	878.33c ± 20.08	0.785b ± 0.003	2.086a ± 0.279	47.51e ± 2.01
L_2_C_2_H_4_	1999.50a ± 18.67	881.70a ± 21.79	2881.19a ± 91.46	398.15a ± 11.38	234.00b ± 20.23	19.37b ± 0.76	1077.00c ± 8.67	849.50d ± 9.47	0.805a ± 0.008	2.170a ± 0.029	46.15b ± 0.52
L_2_C_2_H_5_	1719.08b ± 0.84	799.55b ± 23.36	2518.63b ± 26.36	340.56b ± 1.92	231.33b ± 13.00	17.52c ± 0.09	1003.00d ± 42.57	772.17d ± 15.85	0.799b ± 0.008	2.138a ± 0.066	26.98h ± 1.17
L_2_C_3_H_1_	1844.53b ± 22.57	914.28a ± 32.02	2758.81a ± 54.57	333.57b ± 3.76	191.33d ± 13.58	8.40g ± 0.41	1104.00c ± 0.18	867.00b ± 1.15	0.805b ± 0.002	1.669b ± 0.013	42.36f ± 1.98
L_2_C_3_H_2_	1750.26b ± 12.90	908.51a ± 22.36	2658.77b ± 22.36	318.13b ± 2.89	204.00c ± 26.01	12.33f ± 0.10	1133.67b ± 34.87	912.67b ± 26.86	0.798a ± 0.006	2.272a ± 0.088	40.87f ± 0.01
L_2_C_3_H_3_	1564.22c ± 28.78	833.13b ± 16.16	2397.35c ± 18.34	264.11c ± 1.34	221.33c ± 31.37	18.97b ± 0.35	1168.00b ± 3.43	942.33a ± 16.33	0.809a ± 0.003	2.033a ± 0.104	37.91g ± 0.24
L_2_C_3_H_4_	1516.63c ± 3.35	768.34b ± 17.20	2284.97c ± 19.75	261.51c ± 5.17	209.67c ± 5.24	19.18b ± 1.19	1181.00a ± 9.24	942.17a ± 9.77	0.807a ± 0.005	1.981a ± 0.033	36.60g ± 1.35
L_2_C_3_H_5_	1403.14c ± 29.35	629.18c ± 10.74	2032.32d ± 23.93	241.08d ± 9.90	159.67e ± 9.53	23.26a ± 0.17	1200.00a ± 14.35	946.00a ± 14.35	0.801b ± 0.003	1.693b ± 0.129	62.54d ± 1.36

The same lowercase letters indicate no significant difference in treatments (Scott-Knott test, *p* ≤ 0.05). The colors indicate the magnitude of the observed values, with red representing the lowest values, yellow corresponding to intermediate values, and green indicating the highest values.

**Table 3:** Summary of the analysis of variance for relative water content (RWC), electrolyte leakage (EL), stomatal conductance (*gs*), transpiration (*E*), initial fluorescence (F_0_), catalase activity (CAT), and ascorbate peroxidase activity (APX) in naturally colored cotton under irrigation levels (L) and hydroretentive polymer application (H) at 75 days after sowing in Experiment I.

Source of Variation	DF	Mean Squares						
		RWC	EL	*gs*	*E*	F_0_	CAT	APX
Irrigation levels (L)	1	65.36 *	4231.69 **	0.080 **	9.92 **	300.66 *	1590.96 **	7398.55 **
Hydroretentive polymer (H)	4	624.66 **	1032.28 **	0.043 **	1.48 **	7950.40 **	185.49 **	41,250.68 **
Linear regression	1	22.28 ^ns^	389.29*	0.005 ^ns^	0.99 **	1302.44 **	1680.65 **	25,523.29 **
Quadratic regression	1	40.44 ^ns^	1234.52 **	0.000 ^ns^	1.03 **	814.93 **	6185.93 **	54,930.64 **
Cultivars (C)	2	46.55 ^ns^	217.74 ^ns^	0.019 ^ns^	2.91 **	3803.33 **	111.90 **	39,334.55 **
L × H	4	231.92 **	966.12 **	0.008 ^ns^	0.50 ^ns^	3777.28 **	329.18 **	8803.26 **
L × C	2	231.73 **	534.18 **	0.006 ^ns^	0.09 ^ns^	373.86 **	2764.28 **	20,660.68 **
C × H	8	118.11 **	255.07 ^ns^	0.003 ^ns^	0.87 **	958.09 **	104.01 **	7037.95 **
L × C × H	8	81.92 **	281.12 ^ns^	0.004 ^ns^	0.36 *	677.57 **	210.23 **	2811.51 **
Blocks	2	5.46 ^ns^	973.27 ^ns^	0.002 ^ns^	0.96 ^ns^	446.65 ^ns^	14.08 ^ns^	832.07 ^ns^
Residue	58	12.04	143.79	0.003	0.16	74.66	19.04	740.58
CV (%)		4.79	26.80	23.31	11.90	3.71	16.55	13.91

DF—Degrees of freedom; CV—Coefficient of variation; ^ns^, **, *—Not significant, significant at *p* ≤ 0.01 and *p* ≤ 0.05, respectively.

**Table 4:** Eigenvalues and percentage of total variance explained in the multivariate analysis of variance, with significance probability based on Hotelling’s test (*p*-value) for irrigation levels, hydroretentive polymer doses, and cotton cultivars in Experiment II.

								Main Components		
								PC_1_		PC_2_
Eigenvalues (ʎ)								4.81		2.21
Percentage of total variance (S^2^%)								48.10		22.14
Hotteling test for applied irrigate levels (L)								0.01 **		0.01 **
Hotteling test for cultivars (C)								0.01 **		0.01 **
Hotteling test for hydroretentive polymer (H)								0.05 *		0.01 **
Hotteling interaction test (L × C)								0.01 **		0.05 **
Hotteling test for the interaction (L × H)								0.05 *		0.25 ^ns^
Hotteling test for the interaction (C × H)								0.48 ^ns^		0.01 **
Hotteling test for the interaction (L × C × H)								0.01 **		0.01 **
PCs	Correlation coefficients (r)									
	Chl *a*	Chl *b*	Chl t	Car	*Ci*	F_0_	Fm	Fv	Fv/Fm	TSP
PC_1_	0.72 ***	0.84 ***	0.87 ***	0.76 ***	−0.14 ^ns^	−0.18 ^ns^	0.87 ***	0.85 ***	0.80 ***	0.63 **
PC_2_	0.21 ^ns^	0.32 ^ns^	0.29 ^ns^	0.43 ^ns^	−0.83 ***	0.78 **	0.20 ^ns^	−0.20 ^ns^	−0.55 ***	−0.38 ^ns^

*,**, ***, ^ns^—Significant at *p* ≤ 0.05, *p* ≤ 0.01, *p* ≤ 0.001, and not significant, respectively.

**Table 5:** Mean values of chlorophyll *a* (Chl *a*; μg mL^−1^), chlorophyll *b* (Chl *b*; μg mL^−1^), total chlorophyll (Chl t; μg mL^−1^), carotenoids (Car; μg mL^−1^), intercellular CO_2_ concentration (*Ci*; μmol CO_2_ m^−2^ s^−1^), initial fluorescence (F_o_), maximum fluorescence (Fm), variable fluorescence (Fv), maximum quantum efficiency of photosystem II (Fv/Fm), and total soluble proteins (TSP; mg mL^−1^) as a function of irrigation levels, hydroretentive polymer doses, and cotton cultivars at 77 days after sowing in Experiment II.

Treatments	Chl *a*	Chl *b*	Chl *t*	Car	*Ci*	F_0_	Fm	Fv	Fv/Fm	TSP
L_1_C_1_H_1_	1420.16d ± 31.83	1011.45f ± 0.10	2431.62d ± 31.40	300.57d ± 14.21	250.33b ± 7.227	226.00a ± 0.001	1218.33b ± 8.98	992.33b ± 8.98	0.814a ± 0.001	1.014g ± 0.06
L_1_C_1_H_2_	1436.04d ± 44.61	1060.31e ± 0.15	2496.35d ± 44.61	252.32f ± 5.85	246.00b ± 0.578	217.67b ± 4.338	1201.33c ± 29.19	983.67b ± 27.15	0.819a ± 0.004	0.974g ± 0.03
L_1_C_1_H_3_	1608.45b ± 1.08	1135.47d ± 16.03	2743.92b ± 15.54	292.51d ± 7.38	232.00c ± 0.001	207.67b ± 11.85	1169.33c ± 24.28	961.67c ± 21.35	0.822a ± 0.009	1.044g ± 0.06
L_1_C_1_H_4_	1338.35e ± 16.79	1270.60b ± 23.46	2608.96c ± 34.10	339.21b ± 3.98	211.00e ± 0.001	222.00a ± 0.578	1142.00c ± 0.001	920.00d ± 0.57	0.806b ± 0.001	0.873h ± 0.01
L_1_C_1_H_5_	1345.36e ± 10.23	1109.38e ± 33.59	2454.74d ± 38.29	240.26g ± 9.40	196.00f ± 0.001	233.00a ± 14.45	1127.00 ± 0.001	894.00d ± 14.45	0.793c ± 0.013	1.257e ± 0.21
L_1_C_2_H_1_	1391.41d ± 1.10	1096.86d ± 0.01	2488.27d ± 0.25	295.39d ± 14.79	277.00b ± 0.001	196.00c ± 1.15	1104.00d ± 16.54	908.00d ± 15.58	0.822a ± 0.002	1.060g ± 0.21
L_1_C_2_H_2_	1427.45d ± 0.18	1181.28c ± 17.15	2608.72c ± 17.15	304.86d ± 5.42	243.00b ± 0.001	226.00a ± 4.04	1151.33c ± 28.14	925.33d ± 25.57	0.804b ± 0.004	1.146f ± 0.13
L_1_C_2_H_3_	1482.15d ± 26.43	1206.95c ± 0.20	2689.10c ± 0.86	310.49c ± 5.28	237.00c ± 0.001	231.00a ± 5.78	1163.33c ± 33.42	932.33d ± 37.70	0.801b ± 0.010	1.024g ± 0.11
L_1_C_2_H_4_	1561.47c ± 6.97	1213.10c ± 1.41	2774.56b ± 30.41	318.36c ± 6.90	231.33c ± 0.334	235.33a ± 10.69	1175.67c ± 54.67	940.33c ± 23.02	0.799b ± 0.011	1.148f ± 0.12
L_1_C_2_H_5_	1781.32a ± 32.61	1366.28a ± 10.88	3147.60a ± 45.48	373.05a ± 1.15	195.33f ± 19.07	234.67a ± 2.67	1158.67c ± 82.43	924.00d ± 15.96	0.795b ± 0.015	0.991g ± 0.08
L_1_C_3_H_1_	1610.16b ± 14.30	1184.60c ± 34.98	2794.76b ± 64.28	346.31b ± 10.22	197.00f ± 0.578	238.00a ± 14.43	1183.33c ± 62.24	945.33c ± 26.47	0.798b ± 0.012	1.036g ± 0.18
L_1_C_3_H_2_	1528.58c ± 25.62	1128.45d ± 9.19	2657.03c ± 26.38	315.50c ± 1.86	220.00d ± 0.001	226.00a ± 1.73	1174.33c ± 14.36	948.33c ± 12.93	0.808b ± 0.001	0.867h ± 0.01
L1C3H3	1504.02c ± 10.03	1103.78d ± 44.60	2607.80c ± 121.63	309.97c ± 9.56	252.00b ± 4.377	223.00a ± 0.578	1155.33c ± 66.85	932.33d ± 67.29	0.806b ± 0.012	0.888h ± 0.07
L_1_C_3_H_4_	1470.07d ± 3.12	1032.59e ± 18.53	2502.66d ± 20.39	295.92d ± 8.90	224.67d ± 11.36	234.00a ± 6.93	1095.00d ± 0.001	861.00e ± 6.93	0.786c ± 0.006	0.833h ± 0.02
L_1_C_3_H_5_	1431.29d ± 13.90	1010.47f ± 36.40	2441.77d ± 30.51	269.42e ± 10.59	187.67f ± 11.36	239.67a ± 0.883	1085.00d ± 0.001	845.33e ± 0.88	0.779d ± 0.001	0.964g ± 0.11
L_2_C_1_H_1_	1156.15f ± 10.01	949.58f ± 5.83	2105.73f ± 4.94	203.36g ± 2.53	281.00a ± 19.37	216.67b ± 0.001	1089.00d ± 57.39	860.00e ± 57.39	0.789c ± 0.011	1.292e ± 0.15
L_2_C_1_H_2_	1322.95e ± 3.24	961.31f ± 16.92	2284.26e ± 29.16	220.96g ± 2.98	270.00b ± 8.658	217.33b ± 3.76	1120.00c ± 27.74	902.67d ± 29.66	0.806b ± 0.007	1.581d ± 0.18
L_2_C_1_H_3_	1332.97e ± 13.55	970.19f ± 7.49	2303.17e ± 14.90	225.52g ± 3.58	226.33d ± 0.001	225.33b ± 14.74	1154.33c ± 63.69	929.00d ± 21.66	0.803b ± 0.021	1.725c ± 0.22
L_2_C_1_H_4_	1470.80d ± 23.46	1073.21e ± 4.58	2544.01d ± 45.24	273.59e ± 6.11	211.00e ± 0.001	232.00a ± 4.04	1246.00b ± 0.001	1014.00b ± 4.04	0.826a ± 0.003	1.588d ± 0.16
L_2_C_1_H_5_	1649.96b ± 0.07	1145.18d ± 17.35	2795.14b ± 56.32	296.06d ± 3.43	200.00f ± 0.001	238.00a ± 5.78	1284.00a ± 0.001	1046.00a ± 5.78	0.827a ± 0.005	1.379e ± 0.14
L_2_C_2_H_1_	1146.91f ± 14.10	1014.37f ± 0.97	2161.28f ± 31.97	227.59g ± 4.23	219.00d ± 0.001	242.00a ± 0.001	1082.00d ± 0.001	840.00e ± 0.001	0.776d ± 0.001	1.619d ± 0.11
L_2_C_2_H_2_	1451.66d ± 27.00	995.44f ± 49.41	2447.10d ± 7.52	240.44g ± 11.72	221.33d ± 2.607	213.33b ± 21.52	1012.00e ± 11.56	798.67f ± 11.36	0.790c ± 0.019	2.231a ± 0.24
L_2_C_2_H_3_	1655.20b ± 33.74	1043.41e ± 4.58	2698.61c ± 24.39	283.93d ± 3.43	269.67b ± 4.672	198.67c ± 17.68	978.00e ± 0.001	779.33f ± 17.68	0.797c ± 0.018	1.650d ± 0.11
L_2_C_2_H_4_	1387.30d ± 33.78	845.89g ± 17.60	2233.19e ± 45.22	234.83g ± 3.98	272.00b ± 0.001	216.67b ± 3.84	966.00e ± 0.001	749.33g ± 3.84	0.776d ± 0.004	1.629d ± 0.29
L_2_C_2_H_5_	1326.63e ± 2.87	818.13g ± 0.20	2144.76f ± 2.87	225.30g ± 3.95	201.00f ± 0.001	232.67a ± 2.60	961.00e ± 0.001	728.33g ± 2.60	0.758e ± 0.003	1.915b ± 0.09
L_2_C_3_H_1_	1326.78e ± 89.08	970.23f ± 7.39	2297.01e ± 111.55	232.28g ± 1.01	245.50b ± 9.974	226.00a ± 0.001	1059.0d ± 0.001	833.00e ± 0.001	0.787c ± 0.001	1.381e ± 0.13
L_2_C_3_H_2_	1445.22d ± 76.17	1009.65f ± 4.58	2454.87d ± 120.46	232.79g ± 2.12	247.00b ± 11.38	215.00b ± 0.001	1172.33c ± 24.56	957.33c ± 24.56	0.816a ± 0.004	1.760c ± 0.15
L_2_C_3_H_3_	1465.31d ± 30.74	1048.07e ± 17.83	2513.38d ± 47.58	236.82g ± 10.68	248.00b ± 0.001	221.00a ± 0.001	1180.00c ± 19.07	959.00c ± 19.07	0.813a ± 0.003	1.285e ± 0.11
L_2_C_3_H_4_	1453.60d ± 7.51	1048.57e ± 23.34	2502.16d ± 21.72	242.38g ± 7.76	236.00c ± 0.57	222.33a ± 1.45	1177.33c ± 30.96	955.00c ± 30.70	0.811a ± 0.005	1.784c ± 0.14
L_2_C_3_H_5_	1313.40e ± 17.15	991.98f ± 3.56	2305.38e ± 13.59	251.01f ± 2.37	193.67f ± 0.883	235.00a ± 0.001	1106.67d ± 38.55	871.67e ± 18.55	0.785c ± 0.016	1.443d ± 0.23

The same lowercase letters indicate no significant difference in treatments (Scott-Knott test, *p* ≤ 0.05). The colors indicate the magnitude of the observed values, with red representing the lowest values, yellow corresponding to intermediate values, and green indicating the highest values.

**Table 6:** Summary of the analysis of variance for relative water content (RWC), electrolyte leakage (EL), stomatal conductance (*gs*), transpiration (*E*), CO_2_ assimilation rate (*A*), superoxide dismutase (SOD), catalase (CAT), and ascorbate peroxidase (APX) in naturally colored cotton under irrigation levels (L) and hydroretentive polymer application (H) at 77 days after sowing in Experiment II.

Source of Variation	DF	Mean Squares							
		RWC	EL	*gs*	*E*	*A*	SOD	CAT	APX
Irrigation levels (L)	1	4.01 ^ns^	3434.84 **	0.0000 ^ns^	0.71 ^ns^	49.87 **	4216.17 **	6934.44 **	12,2249.87 **
Hydroretentive polymer (H)	4	522.53 **	950.21 **	0.0002 ^ns^	0.01 ^ns^	67.37 **	2531.74 **	913.81 **	49,796.41 **
Linear regression	1	224.87 **	54.32 ^ns^	0.0005 **	2.60 **	32.34 **	333.51 **	1096.21 **	21,432.72 **
Quadratic regression	1	53.35 **	9.80 ^ns^	0.0045 ^ns^	0.005 ^ns^	2.68 ^ns^	411.02 **	4156.80 **	3873.47 ^ns^
Cultivars (C)	2	514.28 ^ns^	61.01 ^ns^	0.0137 **	10.32 **	109.34 **	67.06 **	183.43 ^ns^	73,612.09 **
L × H	4	235.37 **	294.07 **	0.0021 ^ns^	0.41 ^ns^	4.04 ^ns^	2054.54 **	85.34 ^ns^	17,758.14 **
L × C	2	378.40 **	257.34 **	0.0032 *	0.32 ^ns^	16.87 *	706.17 **	852.50 **	24,343.76 **
C × H	8	205.26 **	205.36 **	0.0088 **	0.19 ^ns^	28.37 ^ns^	1752.82 **	823.85 **	10,531.85 **
L × C × H	8	701.05 **	274.61 **	0.0064 **	0.06 ^ns^	5.54 ^ns^	1003.66 **	258.02 **	22,116.36 **
Blocks	2	3.90 ^ns^	80.34 ^ns^	0.0005 ^ns^	0.07 ^ns^	60.67 ^ns^	140.41 ^ns^	227.74 ^ns^	1963.61 ^ns^
Residue	58	6.02	52.67	0.0012	0.20	5.85	57.38	85.14	1627.86
CV (%)		3.11	14.64	15.85	16.81	15.99	14.74	19.18	19.42

DF—Degrees of freedom; CV—Coefficient of variation; ^ns^, **, *—Not significant, significant at *p* ≤ 0.01 and *p* ≤ 0.05, respectively.

**Table 7:** Description of the treatments analyzed.

	Doses of Water−Retaining Polymer (dm^−3^ of Soil)				
	0.0	1.5	3.5	5.0	6.5
100% of the crop’s water requirement (L1)	L_1_C_1_H_1_	L_1_C_1_H_2_	L_1_C_1_H_3_	L_1_C_1_H_4_	L_1_C_1_H_5_
	L_1_C_2_H_1_	L_1_C_2_H_2_	L_1_C_2_H_3_	L_1_C_2_H_4_	L_1_C_2_H_5_
	L_1_C_3_H_1_	L_1_C_3_H_2_	L_1_C_3_H_3_	L_1_C_3_H_4_	L_1_C_3_H_5_
40% of the crop’s water requirement (L2)	L_2_C_1_H_1_	L_2_C_1_H_2_	L_2_C_1_H_3_	L_2_C_1_H_4_	L_2_C_1_H_5_
	L_2_C_2_H_1_	L_2_C_2_H_2_	L_2_C_2_H_3_	L_2_C_2_H_4_	L_2_C_2_H_5_
	L_2_C_3_H_1_	L_2_C_3_H_2_	L_2_C_3_H_3_	L_2_C_3_H_4_	L_2_C_3_H_5_

L1C1H1 (100% of the crop water requirement, BRS Rubi and 0.0 g dm^−3^ of soil), L1C1H2 (100% of the crop water requirement, BRS Rubi and 1.5 g dm^−3^ of soil), L1C1H3 (100% of the crop water requirement, BRS Rubi and 3.5 g dm^−3^ of soil), L1C1H4 (100% of the crop water requirement, BRS Rubi and 5.0 g dm^−3^ of soil), L1C1H5 (100% of the crop water requirement, BRS Rubi and 6.5 g dm^−3^ of soil), L1C2H1 (100% of the crop water requirement, BRS Jade and 0.0 g dm^−3^ of soil), L1C2H2 (100% of the crop water requirement, BRS Jade and 1.5 g dm^−3^ of soil), L1C2H3 (100% of the crop water requirement, BRS Jade and 3.5 g dm^−3^ of soil), L1C2H4 (100% of the crop water requirement, BRS Jade and 5.0 g dm^−3^ of soil), L1C2H5 (100% of the crop water requirement, BRS Jade and 6.5 g dm^−3^ of soil), L1C3H1 (100% of the crop water requirement, BRS Verde and 0.0 g dm^−3^ of soil), L1C3H2 (100% of the crop water requirement, BRS Verde and 1.5 g dm^−3^ of soil), L1C3H3 (100% of the crop water requirement, BRS Verde and 3.5 g dm^−3^ of soil), L1C3H4 (100% of the crop water requirement, BRS Verde and 5.0 g dm^−3^ of soil), L1C3H5 (100% of the crop water requirement, BRS Verde and 6.5 g dm^−3^ of soil), L2C1H1 (40% of the crop water requirement, BRS Rubi and 0.0 g dm^−3^ of soil), L2C1H2 (40% of the crop water requirement, BRS Rubi and 1.5 g dm^−3^ of soil), L2C1H3 (40% of the crop water requirement, BRS Rubi and 3.5 g dm^−3^ of soil), L2C1H4 (40% of the crop water requirement, BRS Rubi and 5.0 g dm^−3^ of soil), L2C1H5 (40% of the crop water requirement, BRS Rubi and 6.5 g dm^−3^ of soil), L2C2H1 (40% of the crop water requirement, BRS Jade and 0.0 g dm^−3^ of soil), L2C2H2 (40% of the crop water requirement, BRS Jade and 1.5 g dm^−3^ of soil), L2C2H3 (40% of the crop water requirement, BRS Jade and 3.5 g dm^−3^ of soil), L2C2H4 (40% of the crop water requirement, BRS Jade and 5.0 g dm^−3^ of soil), L2C2H5 (40% of the crop water requirement, BRS Jade and 6.5 g dm^−3^ of soil), L2C3H1 (40% of the crop water requirement, BRS Verde and 0.0 g dm^−3^ of soil), L2C3H2 (40% of the crop water requirement, BRS Verde and 1.5 g dm^−3^ of soil), L2C3H3 (40% of the crop water requirement, BRS Verde and 3.5 g dm^−3^ of soil), L2C3H4 (40% of the crop water requirement, BRS Verde and 5.0 g dm^−3^ of soil), L2C3H5 (40% of the crop water requirement, BRS Verde and 6.5 g dm^−3^ of soil).

## Data Availability

The original contributions presented in this study are included in the article. Further inquiries can be directed to the corresponding author.
